# Identification of outcome-related driver mutations in cancer using conditional co-occurrence distributions

**DOI:** 10.1038/srep43350

**Published:** 2017-02-27

**Authors:** Victor Treviño, Emmanuel Martínez-Ledesma, José Tamez-Peña

**Affiliations:** 1 Escuela de Medicina, Tecnologico de Monterrey, Av. Morones Prieto 3000 Pte. Monterrey, Nuevo Leon 64710, Mexico; 2 Department of Genomic Medicine, The University of Texas MD Anderson Cancer Center, Houston, TX 77030, USA

## Abstract

Previous methods proposed for the detection of cancer driver mutations have been based on the estimation of background mutation rate, impact on protein function, or network influence. In this paper, we instead focus on those factors influencing patient survival. To this end, an approximation of the log-rank test has been systematically applied, even though it assumes a large and similar number of patients in both risk groups, which is violated in cancer genomics. Here, we propose VALORATE, a novel algorithm for the estimation of the null distribution for the log-rank, independent of the number of mutations. VALORATE is based on conditional distributions of the co-occurrences between events and mutations. The results, achieved through simulations, comparisons with other methods, analyses of TCGA and ICGC cancer datasets, and validations, suggest that VALORATE is accurate, fast, and can identify both known and novel gene mutations. Our proposal and results may have important implications in cancer biology, bioinformatics analyses, and ultimately precision medicine.

Cancer is a genetic disease characterized by the progressive accumulation of mutations^1^. Recent advances in sequencing technologies are revolutionizing cancer-targeted medicine with their rich characterization of genetic mutations^2^. International efforts, such as the Cancer Genome Atlas (TCGA) and the International Cancer Genome Consortium (ICGC), have been established to scrutinize several cancer types by generating large amounts of cancer genomics data^3^. Nevertheless, due to its intrinsic complexity, there is a need for more advanced and precise methods of analyzing these data to gain a deeper understanding of this deadly disease.

One fundamental problem in cancer genomics is the detection of functional mutations. In this context, the progressive accrual of mutations^1^ and its heterogeneity^4^ have fueled theories of clonal expansion^5^ in which ‘driver’ mutations have functional roles that confer cell fitness advantages, whereas ‘passenger’ mutations are the result of the inherently random mutational process^6^,^7^. The detection of driver genes is challenging, as the observed frequency of gene mutations is relatively low for most of the genes^8^. In addition, the detected gene driver mutations explain only a fraction of mutations per patient^1^,^8^, suggesting that novel methods to detect driver mutations are still required, even though other genetic alterations may also be present (such as copy number, fusions, and epigenetic alterations).

Most of the methods of detecting driver mutations that have been proposed^9^ can be classified according to their main concept into either (*i*) methods that identify recurrent gene mutations or (*ii*) methods that identify the impact of the disrupted protein function. For recurrent gene mutations, methods such as MutSigCV^10^ and OncodriveCLUST^11^ use specific ‘null’ models to estimate background mutation rates and recognize those genes that fail such a model. Indeed, these ideas have also been applied to non-coding regions^12^. In addition, there is a subclass of methods focused on recurrent gene mutations per network modules, such as HotNet2^13^. Contrary, the other methods detect known of putative functional impact of mutations, such as SIFT^14^ and MutationAssessor^15^ that look at disruptions in evolutionarily conserved functional domains, which can be assessed on a variant-by-variant basis. Other methods such as OncodriveFM^16^ examines a set of variants to evaluate whether functional impacts per variant are shifted toward higher impacts.

The abovementioned methods consider mutations from a cellular or molecular viewpoint, wherein the effects that take place locally are assumed to improve the fitness of the tumor cell to its microenvironment. Nevertheless, there are gene mutations that violate the model assumptions, complicating their identification; for example, when the functional effect of a mutation is unknown, or when a mutation is indeed similar to those that are evolutionarily conserved, but that, in the human context, are damaging^17^. On the other hand, model parameters may be over-simplified, such as the replication timing that has been shown to be highly influenced in a cell type-specific manner^18^. In addition, there could be other situations that are more difficult to model, such as mutations appearing in recurrent tumors^19^, tissue invasion or metastasis^20^,^21^,^22^, or co-occurring alterations^23^. Some of these will clearly have an effect on tumor development and time to death, metastasis, or recurrence. Therefore, to overcome some of these limitations, we have adopted here a ‘long-term’ population risk view in which mutations may influence patient survival. This is important, as the precise identification of mutations that are relevantly associated with clinical outcomes could be crucial for treatment and tailoring the precision of medicine^24^.

Generally, the identification of gene mutations associated with survival is accomplished by forming two risk groups, separating those subjects who are carrying the mutation from those who are not. The differences in time to death between these risk groups are used to detect the relevant mutated genes. The statistical significance of this time difference is usually estimated by computing the Log-Rank statistic, followed by the computation of its probability of being zero^25^. Commonly, this p-value is approximated by Gaussian or *χ*^2^ distribution^25^. This procedure, which will hereafter be referred to as the ‘Approximate Log-Rank Test’ (ALRT), assumes that both populations have a similar number of subjects and that this number is large^26^. In cancer genomics, however, these assumptions are generally not met, as the frequency of mutations in a gene across patients is generally low^8^. To address these issues, a precise estimation of the null distribution is required. However, this is challenging, since there is no analytical form of the log rank statistic (to our knowledge) and the number of combinations is astronomically high even for a low number of subjects and mutations, making exact estimations of the null distribution computationally impractical. Therefore, due to the lack of statistical and computational tools, the ALRT is commonly applied, as can be seen in cancer genomics data portals (see *Correlate Clinical vs Mutation* in ‘Clinical Analyses’ from any cancer type within http://firebrowse.org). Recently, a method that accelerates the estimation of the null distribution, ExaLT, has been proposed, in which the number of combinations is reduced under certain circumstances that are controlled by a precision parameter^27^. However, this algorithm is still prohibitively slow, even for moderate population sizes (around n = 200) and precision values. Consequently, there remains a need for fast and accurate methods to estimate the null distribution and the probability of associations between mutated genes and cancer survival time, which may aid the discovery of novel genes and mutations, and provide important insights into cancer biology.

Here, we propose VALORATE (*Velocity and Accuracy for the LOg RAnk TEst*), a novel algorithm that provides, in a timeous manner, a precise and accurate estimation of the empirical null distribution and the probability value of the Log-Rank statistic being zero, regardless of the population size and the fraction of mutations. First, we validate the accuracy and speed of VALORATE for simulated data and a cancer dataset. Then, we apply the algorithm to analyze the gene mutations associated with survival times in 61 cancer datasets, including more than 40 cancer types from TCGA and ICGC, which cover 11,655 and 2,779 cancer samples respectively. We note that, regardless of the method, the significance can be influenced by hypermutated samples. Next, we compare the estimations of VALORATE and the ALRT, suggesting that the ALRT may detect many false positive and false negative genes. As a consequence of these discrepancies, we observe that the proportions of genes associated with low- and high-risk groups are significantly different. We find that genes associated with survival, according to VALORATE, were mostly cancer type-specific. From the identified genes, many are well-known cancer genes, but many others are novel associations. These results appear to be reliable because significant genes are apparently expressed in the mutated tissues and their mutations have high functional impacts. We conclude that VALORATE is a valuable tool for cancer genomics and may be useful for other statistical applications.

## Results

### Validation of the VALORATE algorithm

**Figure 1:**
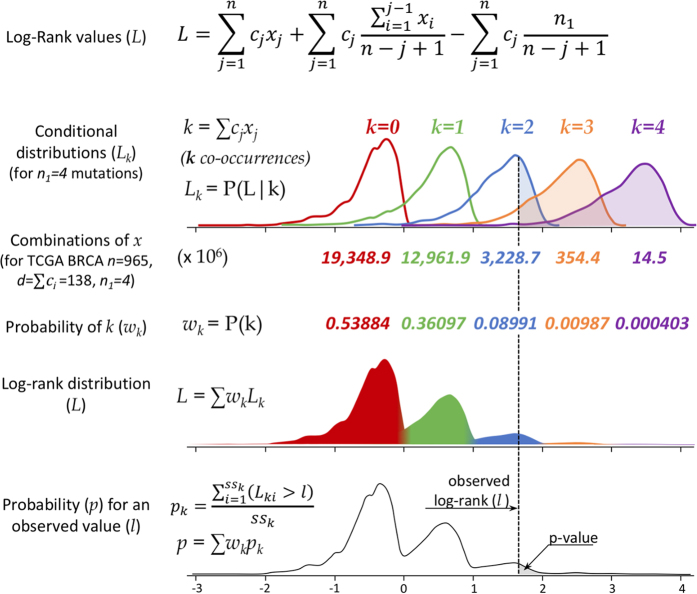
Overview of the VALORATE algorithm. For a dataset having *n* samples, *d* deaths, and a gene having *n*_*1*_ sample mutations coded in the vector *x* of mutated subjects, the conditional distributions *L*_*k*_ are estimated by random sampling *x* over *k*, where *k* is the number of co-occurrences (events that are also mutated). The proportional weight *w*_*k*_ of each *L*_*k*_ can be estimated by the contribution to the total number of combinations, which for a given *k* can be calculated by *C(n* − *d, n*_*1*_ − k)*C(d, k), where *C* is the combination function. The overall distribution is then estimated by a weighted sum on *L*_*k*_. Finally, the p-value for an observed log-rank value in a mutated gene can be estimated by weighting the conditional p values over *k*.

The VALORATE algorithm shown in Fig. 1 (see details in Materials and Methods) is based on the postulate that the log-rank distribution *L* will be highly dependent on *k*, the number of co-occurrences of events and mutations when the number of subjects in one group is very low or, presumably, when there is a highly unbalanced number of subjects between groups. Thus, *L* is a weighted sum of conditional distributions (*L*_*k*_), such as *L* = *∑w*_*k*_*L*_*k*_, where *w*_*k*_ is the fraction of combinations of *k* over all possible combinations. The procedure can be computed rapidly because *L*_*k*_ can be estimated by sampling depending on a *sampling size* parameter (see the methods section and the legend of Fig. 1 for details).

**Figure 2:**
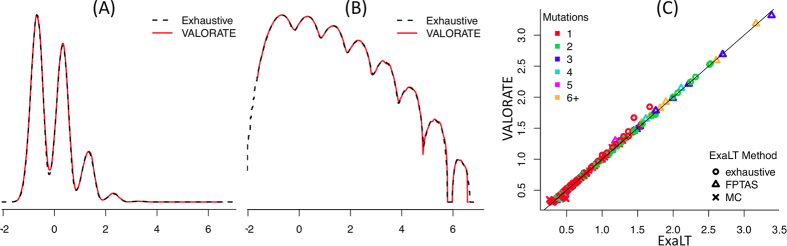
Accuracy of VALORATE. (**A**) Comparison of the exact log-rank distribution for a simulated dataset with the distribution estimated by VALORATE using *ss* = *100,000*. The simulation was estimated by *n* = *100* subjects, *d* = *10* events, and *n*_*1*_ = *7* mutations, which generates 16,007,560,800 combinations. (**B**) Densities in logarithm base 10 to show details in low-density regions (above 3). (**C**) Comparison of the p-values estimated by VALORATE and those estimated by ExaLT for the GBM dataset from Vandin *et al*.^27^ shown in Supplementary Material (TableR.txt file in https://github.com/fvandin/ExaLT). p-values are shown in negative of the logarithm base 10.

To demonstrate the accuracy of VALORATE in the estimation of the log-rank distribution, we used simulations comparing the exact distribution of all possible combinations of the log-rank statistic with the distribution estimated by VALORATE. The simulations were performed under parameter settings whose exact distribution can be computed in sensible computational times. A representative simulation shown in Fig. 2A suggests that VALORATE can accurately estimate the log-rank distribution and that it is consistent in a variety of simulated scenarios (Supplementary Figure 1). As expected, the most extreme statistics of the distribution were not observed due to random sub-sampling (Supplementary Figure 2). This small caveat is not an issue because the estimated *p* values, according to our procedure for these extreme statistic values, would be, correctly, close to 0 and the observed statistics close to these extremes are accurately sampled (Fig. 2B and Supplementary Figure 1B).

To verify the accuracy of the VALORATE procedure in terms of the p-value estimations, we ran some simulations varying the number of subjects per group. The results show that when the assumption of a similar number of subjects in the two groups is met (*n* = *100* subjects and *n*_*1*_ = *50* mutated), as in the ALRT, the p-values estimated by the ALRT and VALORATE are highly similar and highly correlated, independently of the co-occurrence *k* (Supplementary Figure 3). However, when the number of subjects between groups becomes more dissimilar (*n*_*1*_ = *30, 14, or 7*), the differences in p-value estimations become more distinct, which correlates with changes in the symmetry and shape of the overall log-rank distribution. Additionally, the differences in p-value estimations also depend on the number of events co-occurring in the mutated risk group (Supplementary Figure 3). For example, in an extreme case when *n*_*1*_ = *7*, where the number of events in the mutated group was *k* = *0* (so that the seven mutated samples were censored), the ALRT estimated a p-value of 0.15, whereas VALORATE estimated 1.8 × 10^−4^ (Supplementary Figure 4). In contrast, in a case where *k* = *1*, the ALRT estimated a p-value of 3.5 × 10^−6^, whereas VALORATE estimated 0.27. For these estimations, VALORATE used *n*_*1*_* *+* 1* estimated distributions *L*_*k*_, whereas the ALRT used one χ^2^ distribution.

The above results indicate that the VALORATE procedure can accurately (*i*) approximate the exact *L* distribution independent of the shape of the log-rank distribution and (*ii*) calculate the correct p-values, since they converge to the ALRT when *n*_*1*_ is similar to *n/2*. Based on this, we propose that VALORATE is superior to the ALRT when it comes to estimating the probability of the difference between two survival curves, especially in cases where *n*_*1*_ departs from *n/2*.

Then, we evaluated the precision of VALORATE on repeated runs across different values of sampling sizes from 10^3^ to 10^6^. The results show that at *ss* = *10,000*, different runs are almost indistinguishable, indicating high precision (Supplementary Figure 5). Even for *ss* = *1,000*, the shape of the distribution is highly similar to that in *ss* = *1,000,000*, demonstrating that the procedure is consistent and robust. Nevertheless, with a low sampling size, two runs may display slight differences. Although the differences in the estimation of the distribution and, hence, the p-values should be small, it is preferable to use larger sampling sizes to avoid small fluctuations between runs. Therefore, we used *ss* = *100,000* for the cancer data analyses.

To evaluate VALORATE in cancer data, including the estimation of p-values, we compared the calculations against those provided by ExaLT, which is based on three different approaches. The results were strikingly similar between the different methods and numbers of mutations (Fig. 2C), suggesting that the p-value estimations from VALORATE are also accurate in cancer data.

The computation time is an important issue because genomics data are generated at high rates and typical analyses may involve estimations for the available stratifications (e.g. cancer sub-types, hormonal status, histological grades) and within systematic pipelines and data versions. Therefore, we assessed the running time for VALORATE and ExaLT by changing the main parameter associated with accuracy. Despite the fact that both algorithms are different in essence, this test illustrates the timescale needed and how they grow. Table 1 shows that VALORATE runs more than 10,000 times faster than ExaLT; in addition, the running time of VALORATE does not increase drastically.

### Comparison of detected gene mutations

**Figure 3:**
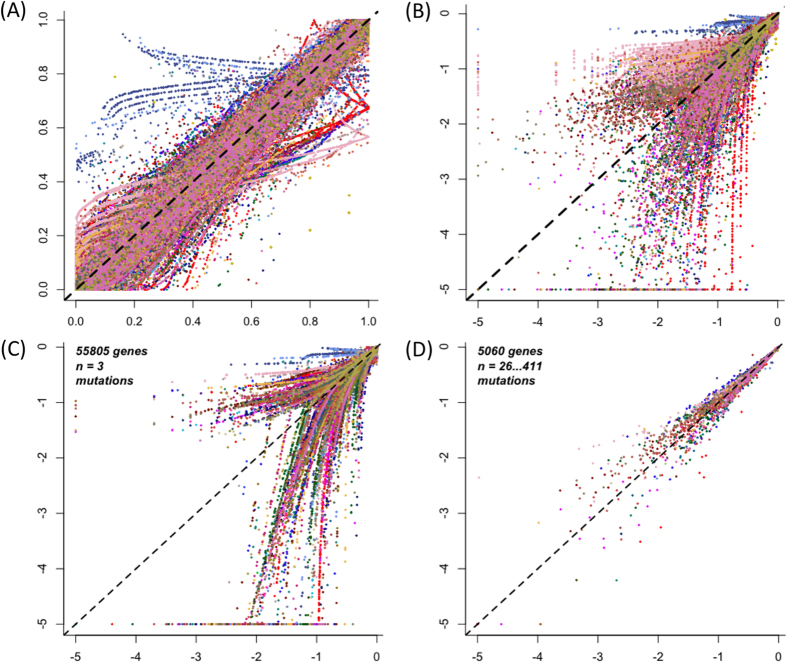
Comparison of the p-value estimations from VALORATE and the ALRT in cancer datasets. (**A**) p-Value estimated by VALORATE (horizontal axis) and the ALRT (vertical axis). (**B**) Same than (**A**) in logarithm base 10 scale to highlight region of significance. Only genes having 4 or more mutations are shown. (**C**) The p-values for genes having 3 samples mutated. (**D**) The p-values for genes having more than 25 sample mutated.

Recent studies have suggested that the ALRT may provide different and false results for the identification of cancer gene mutations associated with outcome^27^. We used VALORATE to compare and analyze the implications regarding the use of a more appropriate test in 61 cancer datasets from the TCGA and ICGC (Supplementary Table 1), covering 14,434 samples, 528,124 genes, and 3,103,054 mutations. We first compared the p-values using the ALRT and VALORATE, for the 152,466 genes that had more than three samples mutated in any cancer. The results demonstrate that, overall, the p-values are highly correlated (Fig. 3A), which further supports the estimations provided by VALORATE. Notwithstanding, a more specific analysis of the most significant region (p < 0.1) shows that the estimations can differ markedly (Fig. 3B). Only 148 genes (0.097%) showed p-values < 0.001 in both tests. At p < 0.01, the ALRT appears to call 1.8 times more genes as significant than does VALORATE. However, we observed certain differences across cancer datasets (Supplementary Figure 6). For example, some cancer types showed more detections in the ALRT, such as breast cancer (BRCA), while others showed more detections in VALORATE, such as uterine corpus endometrial carcinoma (UCEC). We then observed that the differences in p-value estimations between the ALRT and VALORATE are specific for genes with a low number of mutations (Fig. 3C,D), where the ALRT assumptions are not met. Indeed, the differences in p-value estimations decrease with an increasing number of mutations (Supplementary Figure 7). This indicates that, as the simulations suggested, the use of the ALRT estimation is progressively detrimental when reducing the number of mutations in cancer data.

### Identification of outcome-associated driver mutations in cancer

**Figure 4:**
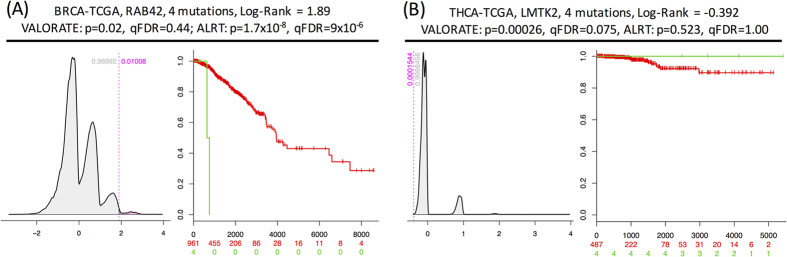
Examples of differences in p-value estimations. (**A**) RAB42 in BRCA from TCGA. (**B**) LMTK2 in THCA from TCGA.

The results shown above may have important implications for cancer genomics, as they raise the possibility that other genes can be identified and that some of the genes previously identified using the ALRT could be suspicious. Therefore, we selected the most significant genes after correction by false discovery rate (FDR < 0.333). We observed large differences in the selected number of genes between VALORATE and the ALRT (Supplementary Figure 8A); only 7% of the genes were identified in both tests, or 34% when considering the rank of top genes (Supplementary Figure 9). Two examples of such discrepancies are shown in Fig. 4 for the genes RAB42 in breast cancer and LMTK2 in thyroid cancer. RAB42 is the most significant gene reported by the TCGA in BRCA (p = 1 × 10^−8^, q = 9 × 10^−6^, http://firebrowse.org, doi:10.7908/C10Z72M8); however, in VALORATE its significance is marginal (p = 0.02) and not selected after FDR correction (q = 0.44). Conversely, the LMTK2 in THCA, which is significantly mutated by frequency using MutSigCV^10^ from TCGA but not associated with time to death using the ALRT (http://firebrowse.org, doi:10.7908/C1542N2H), is the most significant mutated gene in THCA when using VALORATE (p = 0.00026, q = 0.075). All these results suggest that VALORATE can identify genes that are missed by the ALRT and mark genes whose association with survival can be spurious, which contributes important insights in cancer biology.

Nonetheless, we observed an apparent excess of significant mutations in uterine corpus endometrial carcinoma, bladder, and colon (China) (UCEC, BLCA, and COCA-CN respectively) that had more than 200 genes associated with survival, according to VALORATE (Supplementary Figure 8A). The number of significant genes was weakly associated with dataset characteristics such as the number of samples, events, or censoring (Supplementary Figure 10). Instead, it was clearly associated with hypermutated samples (Supplementary Figure 11), which represent a minor proportion of samples with an exacerbated number of mutations^28^. This bias could not be observed using the ALRT in UCEC, as there were no significant genes after FDR correction. However, in BRCA, where the ALRT detects many significant genes and VALORATE does not, the same issue arises when using the ALRT (Supplementary Figure 11). Such a result suggests that hypermutated samples may bias the survival analyses of mutated genes. To explore this further, we re-analyzed all datasets, removing the top 5% most mutated samples. The results show a substantial reduction of detected genes (Supplementary Figure 8B), postulating that hypermutated samples do indeed influence the selection of many genes in several cancer types (except gliomas). Therefore, for further analysis, we used a refined criterion to remove hypermutated samples, avoiding the removal of samples with few mutated genes (Supplementary Figure 12 and Materials and Methods).

### Significant genes and risk assessment

**Figure 5:**
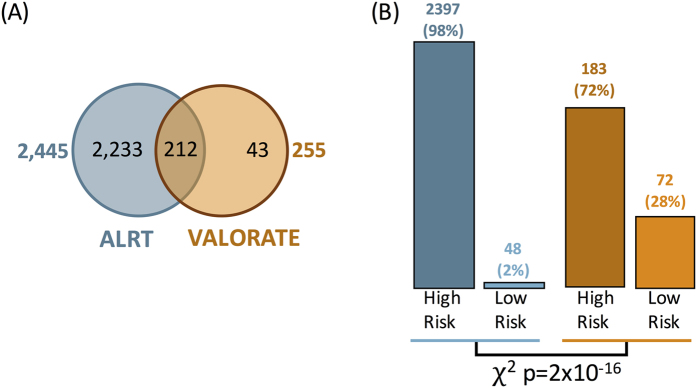
Comparison of significant genes and associated risk. (**A**) The genes detected in two methods. (**B**) The number of genes associated with high- and low-risk.

After removing hypermutated samples, and using an FDR = 0.333, the ALRT calls 2,445 genes significant while VALORATE determines only 255 (Supplementary Figure 13). This decrease is similar across different cancer types. The only exception was Gliomas, in which we observed 164 significant genes with an association, yet the mutations were not apparently related to hypermutated samples (Supplementary Figure 14). From the significant genes, 212 were detected in both tests (Fig. 5A). Interestingly, the risk associated with genes was different between tests (Fig. 5B, χ^2^ test = 2.2 × 10^−16^). In the ALRT, only 2% of identified genes were associated with low risk, whereas in VALORATE the low-risk genes constituted 28%. Overall, in VALORATE, the median hazard ratio was 4.2 for high-risk genes and 0.04 for low-risk genes (several low-risk genes do not include deaths). We observed more low-risk gene mutations in cervical squamous cell carcinoma, esophageal carcinoma, glioblastoma, ovarian cancer, stomach-esophagus cancers, thyroid cancer, uterine corpus endometrial cancer, and a few others (CESC, ESCA, GBM, OV, STES, THCA, and UCEC respectively; see Supplementary Figure 15). These findings may have implications for cancer biology as, apart from a few exceptions such as IDH1 in gliomas, most coding mutations detected so far in cancer have been associated with decreasing survival rates when using the ALRT^29^. In addition, these results may also assist the design of low-risk biomarkers in other cancer types.

### Significant genes across cancer types

**Figure 6:**
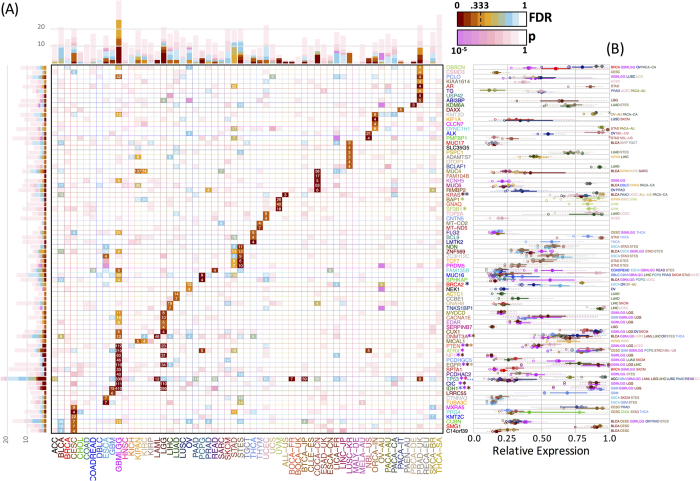
Significant genes across cancer types detected by VALORATE. (**A**) Map of genes detected (vertical) across cancer types (horizontal). The color shades (from brown to orange and cyan) in each cell correspond to FDR q-value when less than 0.9 in which case the number of mutated samples is also shown. In cases where FDR > 0.9, the p-Value is shown in shades of pink. The bars show the accumulated number of cancer types for genes (left bars) or genes for cancer types (top bars). For Gliomas (GBMLGG) and Low-Grade Gliomas (LGG) only the top 10 genes are shown. The genes marked with an asterisk (*) have been reported as significant in FireBrowse (http://firebrowse.org). (**B**) Gene expression levels of the significant genes in those cancer types whose FDR > 0.9 and whose gene expression values were available. The filled circles represent the median (50%) of the expression level and the continuous line the 25% and 75% using all samples. The crossed circles represent the median (50%) of the expression level and the dotted line the 25% and 75% from the mutated samples. The smaller hollow circles represent the lowest expression value from the mutated samples.

Certain genes, such as TP53, PIK3CA, PTEN, KRAS, ARID1A^8^, and others (see www.tumorportal.org) are significantly mutated in several cancer types. As the results shown above suggest that other genes associated with survival can be identified using VALORATE, we compared the significant genes across cancer types (Fig. 6). At FDR = 0.333, only TP53 and MUC4 were found to be significantly associated with survival in at least two ‘distinct’ cancer types (TP53 to GBMLGG, LAML, BOCA-FR, BTCA-JP, PRAD-UK, and MUC4 to COCA-CN, KICH, and KIPAN). TP53 was marginally significant (FDR > 0.33 and p < 0.05) in many other cancer types, while MUC4 was in a few others. Interestingly, ATRX, which is well known in glioblastomas^30^, was significant in gliomas, neuroblastoma, and pheochromocytoma/paraganglioma, all of which are related to the nervous system. Some genes appear to be significant in one cancer type and marginally significant in others. Extreme cases are MUC16, MUC17, PCLO, and DNMT3A, which are marginally significant in a handful of other types. A few genes appear significant in datasets that are composed of similar cancer types, such as gliomas (GBMLGG, which is composed of glioblastoma and low-grade gliomas), stomach-esophagus (STES), and kidney (KIPAN). Apart from these rare exceptions, the majority of the genes seem to be cancer type-specific (Fig. 6) even when the significance assumption is relaxed (Supplementary Figure 16). Remarkably, we did not observe a significant survival association with other well-known cancer genes, such as PIK3CA (lowest p = 0.007, q = 0.5 in UCEC), BRAF (lowest p = 0.028, q = 0.98 in KIRP), and FBXW7 (lowest p = 0.018, q = 0.99 in MELA-AU). From the 77 genes in Fig. 6, only 12 were also significant in the TCGA systematic analyses when using the ALRT. Overall, these results suggest that association with survival is a different feature to mutation frequency, supporting the exploration of association with a broader set of mutated genes, rather than only those detected by frequency or functional impact.

### Functional validation of significant genes

In this study, more than 50 genes were only significant in VALORATE and can be considered novel associations (Fig. 6). In addition, many significant genes were highly ranked in VALORATE and obscured by their rank when using the ALRT (Supplementary Figure 9). This led us to question whether the significant genes may have plausible functional roles. To explore this, we devised two tests for gene expression and functional impact of mutations. For the first, we reasoned that if a mutation in a coding gene is associated with survival, the gene should be expressed. Figure 6 shows that most of the significant genes are expressed at some level. While some show expression levels of around 15% (relative to their cancer), the levels across tissues are highly consistent (for example TP53, MUC17, FLG2, TUBA3C), suggesting a common functional level of expression. Additionally, the expression of mutated samples is highly similar to those observed in their cancer types.

We also tested the possible functional impact associated with the mutations of significant genes that may affect active sites, interactions with other proteins, and 3D folding. This can be corroborated by comparing the non-synonymous mutations against conserved sequences in homolog proteins. For this, we used MutationAssesor^15^, which summarized the functional impact into neutral, low, medium, and high impact. We observed that the mutations in the identified genes had significantly higher impact categories than randomly chosen mutated genes from the same cancer types and a similar number of mutations (Supplementary Figure 17).

All of the above results suggest that, in general, coding mutations of the identified significant genes can be functional.

## Discussion

A fundamental problem in cancer genomics and precision medicine is the determination of genomic alterations that may be associated with survival times. The selected alterations would then form the foundation for research studies into biological mechanisms, drug discovery, and possible treatments. Such a choice is, however, challenging because most of the observed alterations are present in a low number of patients, there are thousands of alterations to test, and the associations need to be tested in several subject strata (grades, hormonal status, molecular subtypes, and so on). It has recently been shown that the statistical approximations used for this identification in cancer genomics are inaccurate^27^. The failure is basically due to the low number of patients presenting a specific alteration, which generates severely unbalanced population sizes. Although an accurate tool has been recently proposed^27^, it is prohibitively slow to compute in practice. In this work, we revisited the problem of estimation and proposed VALORATE, a novel estimation procedure that is independent of the number of alterations. We showed through simulations that VALORATE is fast, precise, and accurate. In comparison with another method, VALORATE also provided accurate p-values in cancer data. We also demonstrated the precision VALORATE when comparing largely unbalanced populations; at the same time, it is broadly similar to the ALRT when the populations are balanced. Therefore, VALORATE could be utilized in both situations. This should facilitate its use and implementation in current bioinformatics pipelines (see Code section in Methods).

We proved that the ALRT generates poor results under unbalanced populations (Supplementary Figure 3). This is in agreement with previous results^26^,^27^ and explains the notable differences in the number of genes called significant between the ALRT and VALORATE across cancer types. In addition, our simulations (Supplementary Figure 3) demonstrated that the ALRT overestimates the significance for higher values of co-occurrences (*k*) and underestimates the significance for lower values of co-occurrences. This is presumably caused by the lack of symmetry and non-zero centering of the distribution. The underestimation of the significance in the ALRT for lower values of co-occurrences *k* explains why it was unable to call low-risk genes as significant (low-risk genes are characterized by co-occurrences *k* close to zero, due to the low number of deaths). Further simulations confirmed that this pattern is accentuated to a greater degree for fewer mutations and a lower percentage of events independently on the number of samples (Supplementary Figure 18). In addition, a preliminary power analysis suggests that VALORATE shows better performance for low-risk genes but is more conservative for high-risk genes (Supplementary Figure 19).

We also highlighted a couple of examples where there were differences between the estimations of significance between VALORATE and the ALRT (Fig. 4). In this context, our results, which show precise estimations of the exact log-rank distribution, suggest that the ALRT is biased. Nevertheless, the lack of a gold standard or experimental validation of well-known mutated genes associated with survival in which VALORATE and the ALRT clearly differs makes it difficult to assess which procedure performs better. In this context, the results show that the use of VALORATE could guide these kind of experimental validations.

Using VALORATE, we identified that hypermutated samples may bias the estimation of p-values. This issue is related to, at least, two factors. First, VALORATE is a univariate procedure and suffers from its same caveats, for example it tests the alterations in one gene (or locus) at the time while being blind to other alterations. Second, the biology of the cancer-mutation-patient-survival relationship is complex. In UCEC, for example, hypermutated samples exhibit high survival times and are censored; in contrast, in BRCA, they appear as poorer survival. To address this, we first removed the top 5% most mutated samples to demonstrate their impact; then, we refined the criteria avoiding the unnecessary removal of samples to yield fairer estimations. The results produced in this way seem to be independent on hypermutated samples. Despite these steps, the decision over whether to remove samples, including how many and which, deserves further attention.

Regarding datasets that are generated by a union of cancer types such as stomach-esophagus (STES), colorectal (COADREAD), pan-kidney (KIPAN), and others, we observed similar p-value estimations of the mutated genes in individual datasets compared to these merged datasets. However, we observed 164 significant genes in gliomas (GBMLGG), while only 19 and 2 genes were significant in low-grade gliomas (LGG) and glioblastomas (GBM) respectively. This could be related to the fact that LGG exhibits higher survival rates than GBM, so that genes that are more frequently mutated in LGG or GBM, and show some kind of correlation with survival, would likely be significant in GBMLGG. Additionally, we noted that genes that are significant in GBMLGG are not necessarily significant, or even marginally in LGG or GBM. After revision (Supplementary Figure 20), the higher significance in GBMLGG seems to be more related to differences in mutation frequency than to a gain of power, which suggests that significance in GBMLGG is inflated.

A surprisingly large number of cancer types do not show significant gene mutations. It is possible that the small sample size could affect some cancer datasets. For example, in papillary thyroid carcinoma (THCA-SA), we analyzed only 15 samples. In spite of this hypothesis, in other cancers such as breast, bladder, and skin cancer (BRCA, BLCA, and SKCM), no significant genes were found to be associated with survival time at FDR = 0.333, even though these datasets included more than 900, 400, and 340 samples respectively. This indicates that more focused analyses in different strata are required for these cancer types to determine whether there are genes associated to survival. Another example is CLLE-ES, where no significant genes were found using 218 patients, but some of the top ones (EGR2, ASXL1, NOTCH1, POT1, and NXF1) have been reported recently^31^ using more than 450 patients. Accordingly, it is likely that we are detecting fewer genes.

As a proof of concept, we focused on coding mutations; however, it is known that copy number alteration (CNA) is also related to survival^29^,^31^. Therefore, further analyses should also focus on CNA data.

Some mutational biomarkers have been proposed for a handful of cancer types^31^. In this context, we find that certain genes previously identified using the ALRT may change their significance; that others may escalate in rank; that a considerable proportion of genes provide low-risk odds; and that hypermutated samples may influence the identification. These results suggest that novel or refined cancer biomarkers can be identified.

Based on our simulations and analysis of cancer data, we demonstrated that VALORATE is fast, precise, and accurate in its estimation of the null distribution and the p-value of the difference between two survival curves even in cases when the numbers of subjects in survival groups are highly unbalanced. We conclude that VALORATE is a novel and useful tool in cancer genomics and other statistical analyses.

## Methods

### The VALORATE algorithm

It is assumed that there are two groups of individuals and that, for each patient, we know their follow-up time and whether that time represent an event (such as death, metastasis, recurrence) or not (censored). In addition, *n* represents the total number of individuals, *n*_*1*_ the individuals in the mutated group, and *d* the total number of events. There would then be *r* distinct ordered times and *j* = *1*..*r* represents each of these times. Let *R*_*j*_ be individuals at risk that have not yet presented the event and *R*_*1j*_ those at risk for the mutated group. In each time *j*, there would be *O*_*j*_ events (zero or more), and *O*_*1j*_ events for the mutated group. Under the null hypothesis of no difference between groups, *O*_*1j*_ is hypergeometric; therefore, the expected number of events in the mutated group is *E*_*1j*_ = *R*_*1j*_ * *O*_*j*_*/R*_*j*_^25^. The log-rank statistic is then the sum of differences between the expected and the observed number of events^25^, as:


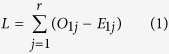


Under certain assumptions (high number of samples, not so few events, and similar group sizes^26^), the mean of *L* is zero, and its variance is:


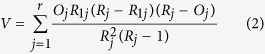


Accordingly, a *L*^*2*^*/V* follows a χ^2^ distribution with one degree of freedom, and this fact can be used to estimate the p-value of *L* being zero, which is equivalent to no difference in the survival curves. Nevertheless, the χ^2^ approximation yields biased estimations when *n*_*1*_ ≪ *n/2 *27, which is the case in cancer genomics. To achieve accurate estimations of the probability of the two survival curves being equal when *n*_*1*_ ≪ *n/2*, we must first rewrite Equation (1) in terms of events ranked by time and their corresponding mutated group^27^, as follows:


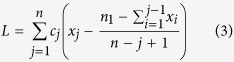


where *c*_*j*_ = *1* is the indicator of an event (death, recurrence, metastases) or *c*_*j*_ = *0* for censored observations (events not yet observed) ordered by time for the *n* subjects, *x*_*j*_ = *1* for subjects that are included in the mutated group, or *x*_*j*_ = *0* for those who are not mutated. In this notation, *n*_*1*_ is equal to *∑x*_*j*_, the number of subjects mutated. To estimate the density of the test statistic *L*, we rearrange Equation (3) as follows:


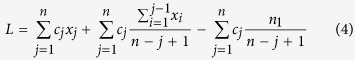


where it is more evident that when *n*_*1*_ ≪ *n/2*, the log-rank statistic *L* should depend strongly on the left term, *k* = 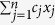, the number of co-occurrences, which represents events (*c*_*j*_ = *1*) that are also mutated (*x*_*j*_ = *1*). In contrast, when *n*_*1*_ is close to *n/2, k* should be close to half the number of events. The middle term also depends on *x*_*j*_ but is more robust to precise positions of *x*_*j*_ = *1* than the left term, which is highly dependent on the positions of *x*_*j*_ = *1*. The right term, let be *s*_*1*_, is constant. From Equation (4), it follows that *k-s*_*1*_ < *L* < *k* for a given value of *k*. This observation is important because it highlights that the overall log-rank distribution can be seen as a mixture of distributions that depend on the number of co-occurrences *k*. The number of combinations for each co-occurrence *k*, although highly variable, can be easily calculated. To estimate a p-value, the relative proportion of combinations of the co-occurrences *k* is used to weight the relative contribution of the middle term to the overall distribution. Additionally, the distribution of *L* conditional to a specific value of *k (L*_*k*_ = P(L|k)) can be estimated by random sampling (permutations) instead of a non-conditioned all-combinations approach as used in other methods^32^. Consequently, the VALORATE algorithm (Fig. 1) estimates the distribution of *L* by a weighted sum of conditional distributions *L*_*k*_ for specific values of *k* such as *L* = *∑w*_*k*_*L*_*k*_, where *k* varies from 0 to *min(n*_*1*_*, d*) and *w*_*k*_ is the proportional contribution of the combinations for particular co-occurrences *k (w*_*k*_ = *C(n* − *d, n*_*1*_ − *k*) * *C(d, k*), where *C* is the combination function). Finally, the probability of a specific observed statistic, *L*_(*gene*)_, is estimated by the area of the right (or left) fraction. The area is easily estimated by counting the random samples of each *L*_*k*_ that are greater (or lesser) than *L*_(*gene*)_ multiplied by its corresponding weight *w*_*k*_. Ties can be broken by random-sampling the ***c*** vector in tie positions during the estimation of *L*_*k*_, only in tie positions containing mixtures of events and censored observations. During the estimation of the p-value, we estimate the average log-rank statistic permuting tie positions.

The main parameter for VALORATE is the total number of samples (*ss* = *sampling size*) used for the estimation of the whole distribution. For each value of *k*, the sampling size *ss*_*k*_ is obtained by weighting *ss* with respect to the probability of observing *k* (or a minimum of sampling, *ss*_*min*_, or all combinations if *ss*_*k*_ is more than half of the number of combinations for *k*). We commonly use *ss* = *100,000* and *ss*_*min*_ = *1,000*. For each cancer dataset, this procedure was used to obtain *L* for each observed value of *n*_*1*_ (usually for *n*_*1*_* *>* 3* or *n*_*1*_ = *3* in special cases; see the following sections). The p-values have to be multiplied by a factor of 2 for two-tailed tests, which were used for comparisons with the ALRT.

### Cancer mutation data

The mutation and clinical data were obtained from data portals: TCGA (https://tcga-data.nci.nih.gov), specifically from the FireBrowse interface (http://firebrowse.org/), and ICGC (https://dcc.icgc.org). Some ICGC data was accessed through the data access compliance office application DACO-1030211. A summary of the data used is shown in Supplementary Table 1. Overall, 61 cancer datasets were analyzed (35 from TCGA and 26 from ICGC), covering 12,428 cancer samples and 2,772,613 gene mutations (a gene may be mutated more than one time per sample). Only mutations carrying a clear coding effect (missense, nonsense, frame-shift, insertions, deletions, and splicing changes) were used avoiding mutations in introns and untranslated regions.

### Simulations of survival data and mutations

To determine the accuracy and precision of VALORATE, we performed simulations to generate random ***c*** vectors for specific values of *n, n*_*1*_, and *d*. All possible values of ***L*** were calculated, corresponding to all possible combinations of the ***x*** vector. In this way, the *exact* distribution of *L* was obtained and compared to the distribution estimated by VALORATE. We used parameter settings that help to illustrate the comparisons avoiding those combinations of parameters that will need very large computational times.

### Performance analyses

To evaluate and compare the running time of VALORATE, we used the glioblastoma dataset reported in the Supplementary Data from Vandin *et al*.^27^. Specifically, we used the file tableR.txt obtained from https://github.com/fvandin/ExaLT. The p-value estimations used for comparisons with VALORATE in Fig. 2 used all genes. From the three p-values (LEFT, RIGHT, SUM) provided by ExaLT, we used the closest in comparison to VALORATE. The estimations of CPU-time were performed for four genes only, which had the largest number of mutations and were estimated by the ExaLT algorithm (marked as FPTAS) using the default parameters. The genes used were PIK3R1, IDH1, ERBB2, and SYNE1, having 12, 10, 9, and 7 mutations respectively.

### VALORATE analyses

For the simulations, the *ss* parameter used was specified in each particular experiment; *ss*_*min*_ was *1,000* unless otherwise specified. For the cancer data analyses, we used *ss* = *100,000*. We focused on genes that had mutated in more than 4 or 4% of the patients. Therefore, for 46 cancer datasets that had 75 or more subjects with survival data, only genes that had mutated in four or more subjects were used. For the 15 cancer datasets with 74 or fewer subjects, only genes that had mutated in three or more subjects were used.

### Selection of hypermutated samples

The range of mutation rates per cancer type is dependent on the particularities of each cancer type^10^. For instance, the median of the number of mutated genes of neuroblastoma (NBL-US) and thymus cancer (THYM) were 1 and 9 respectively, while this number in bladder (BLCA) and melanoma (MELA-AU) was 169 and 343 (Supplementary Figure 12). Accordingly, to avoid removing samples in cancer types that had few mutations, hypermutated samples were removed if they had more than 500 mutated genes, were within the top 5% of most mutated samples, and the number of mutated genes was larger than the median plus four times the median absolute deviation. The specific number of samples removed per cancer type is shown in Supplementary Figure 12.

### Functional analyses of mutated genes

To validate whether the significant genes obtained by VALORATE could have a functional effect, we performed two analyses. First, we reasoned that a significant gene would likely be expressed to exert a functional role. Thus, we obtained the gene expression levels from TCGA or ICGC data portals of available cohorts to assess the overall relative expression of the significant genes, including the comparison between all subjects and those mutated. Microarray or RNA-Seq data were used (Supplementary Table 1). Second, we considered that significant mutations should be more ‘damaging’ than their random counterparts. Therefore, we used MutationAssessor^15^ to qualify the level of the functional impact of mutations. This tool classifies mutations according to evolutionary conservation patterns of affected amino acids in homolog proteins.

### Code

The VALORATE code was mainly implemented in R, with some portions in C. VALORATE is freely available for download and use under a general MIT license. Instructions for use, examples, and additional information can be found at https://github.com/vtrevino/valorate.

## Additional Information

**How to cite this article:** Treviño, V. *et al*. Identification of outcome-related driver mutations in cancer using conditional co-occurrence distributions. *Sci. Rep.* **7**, 43350; doi: 10.1038/srep43350 (2017).

**Publisher's note:** Springer Nature remains neutral with regard to jurisdictional claims in published maps and institutional affiliations.

## Supplementary Material

Supplementary Figures

Supplementary Table 1

Supplementary Table 2

## Figures and Tables

**Table 1:** Running time for the probability calculation of 4 genes in two algorithms.

Accuracy	VALORATE			ExaLT (FPTAS)		
	Parameter (ss)	CPU Time (sec)	Time Growth	Parameter (af*)	CPU Time (sec)	Time Growth
**Low**	1,000	0.10		1,000	563	
**Modest**	10,000	0.15	1.5×	100	710	1.3×
**Good**	100,000	0.37	2.5×	10	2,405	3.4×
**High**	1,000,000	2.24	6.0×	1	34,910	14.5×
**Extreme**	10,000,000	14.28	6.4×	—	—	

^*^Approximation factor.
