# Supplementary material for: Identification of outcome-related driver mutations in cancer using conditional co-occurrence distributions
Source: Sci Rep. 2017 Feb 27;7:43350. doi: 10.1038/srep43350 (PMC5327384; doi:10.1038/srep43350)
Supplement: Supplementary Figures [file srep43350-s1.pdf]

Supplementary Figures for *"Identification of outcome-related driver mutations in cancer using conditional co-occurrence distributions"*

Victor Treviño*[1], Emmanuel Martínez-Ledesma [2], José Tamez-Peña [1]
[1] Escuela de Medicina, Tecnologico de Monterrey, Av. Morones Prieto 3000 Pte. Monterrey, Nuevo Leon 64710, Mexico.
[2] Department of Genomic Medicine, The University of Texas MD Anderson Cancer Center, Houston, TX 77030, USA.
* corresponding author.

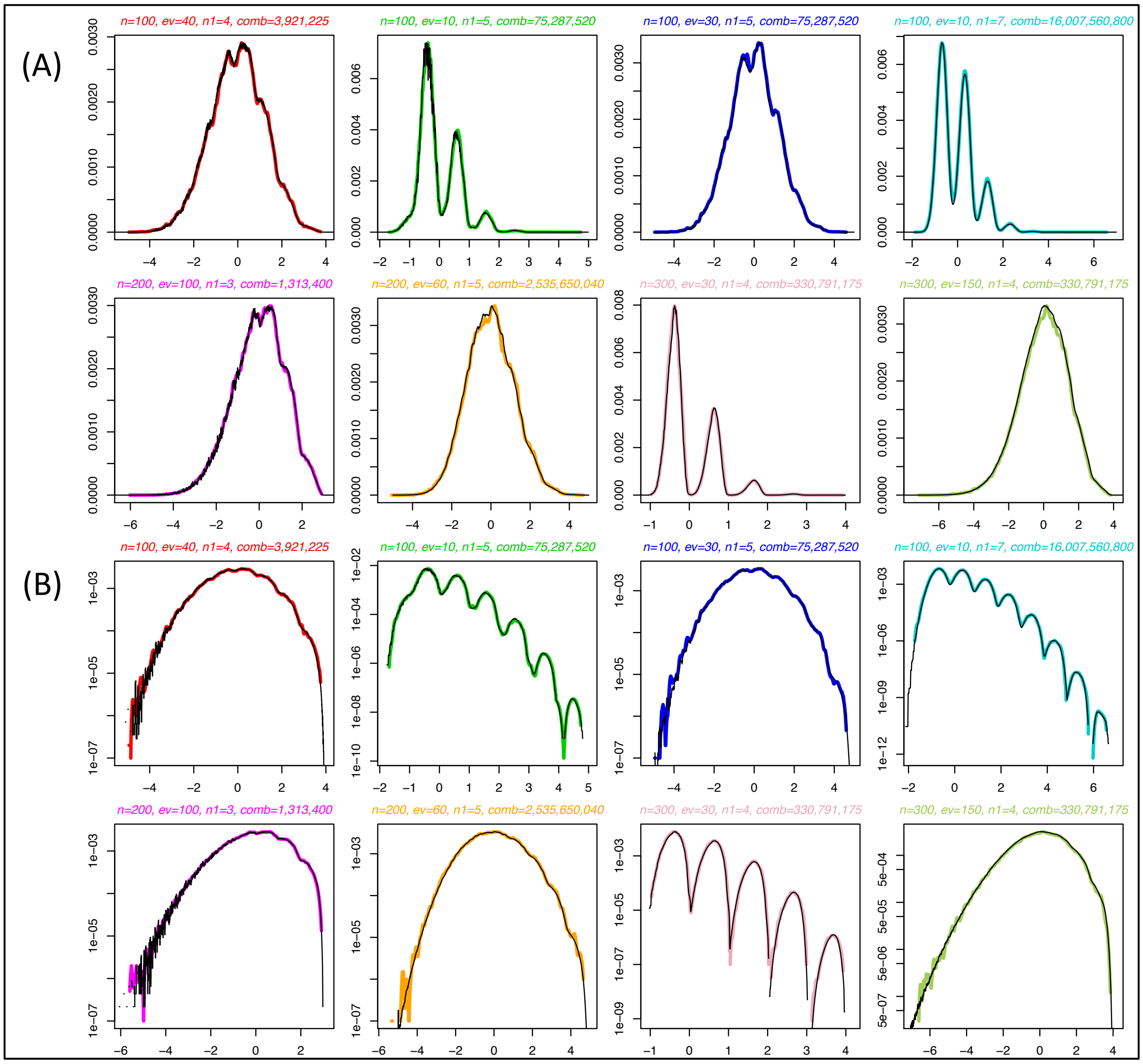

Supplementary Figure 1. Comparisons of exact and estimated log-rank distribution for varied simulations. The black line represents the exact distribution whereas the colored lines show the distributions estimated by VALORATE. The values of *n, d (ev), n1,* and a total number of combinations (*comb)* is included in the top of each panel. The top 8 panels correspond to the distribution of 8 simulated scenarios shown in nominal units. The bottom 8 panels display corresponding distributions in logarithm base 10 scale to highlight local modes of low density.

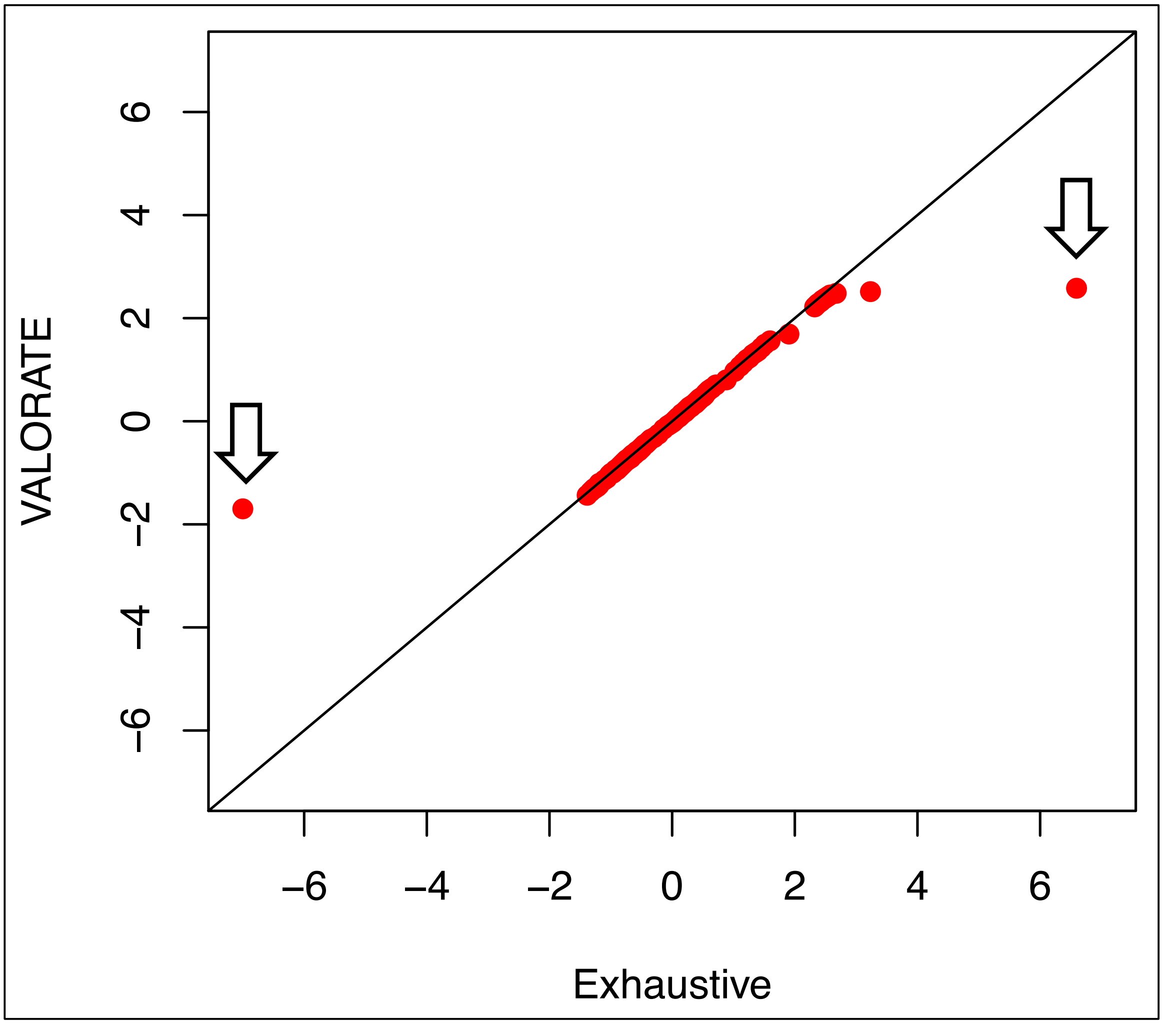

Supplementary Figure 2. QQ plot comparison of distributions. The distributions correspond to the simulation shown in Figure 2 of the main paper. The exhaustive distribution is shown in horizontal axis while the VALORATE distribution is shown in the vertical axis. Each dot corresponds to the value of the distribution from 0% to 100% in increments of 1%. The extreme dots around +/- 6 marked with arrows were seen in the exhaustive calculation but not observed in the random sampling of VALORATE, which is expected due to random nature of the sub-sampling process.

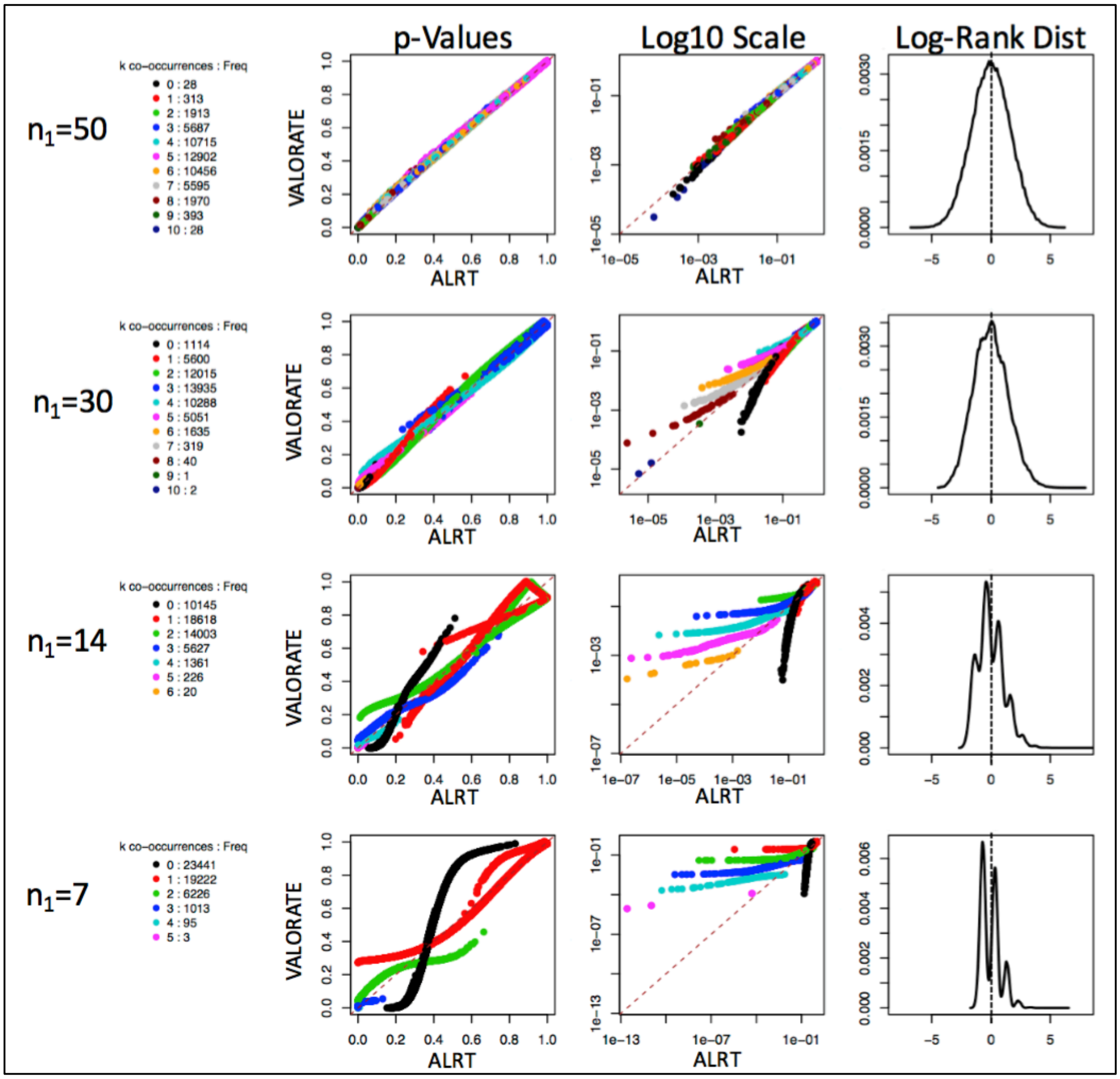

Supplementary Figure 3. Comparisons of p-value estimations. Each row of panels shows a specific simulation varying n_1={ 50, 30, 14, 7 } respectively randomizing the mutational group (*x* vector) 50,000 times and using *n=100* subjects and *d=10* events. The left column shows the observed distribution of *k* co-occurrences (death and mutations), followed by the p-value estimations in linear and logarithmic scales, and the overall *L* distribution estimated by VALORATE. The ALRT p-values are shown in the horizontal axis whereas the VALORATE p-values are shown in the vertical axis. Note that p-value differences are dependent on n_1 and *k*. Some co-occurrences were missing within the 50,000 random vectors in n_1=14 and n_1=7.

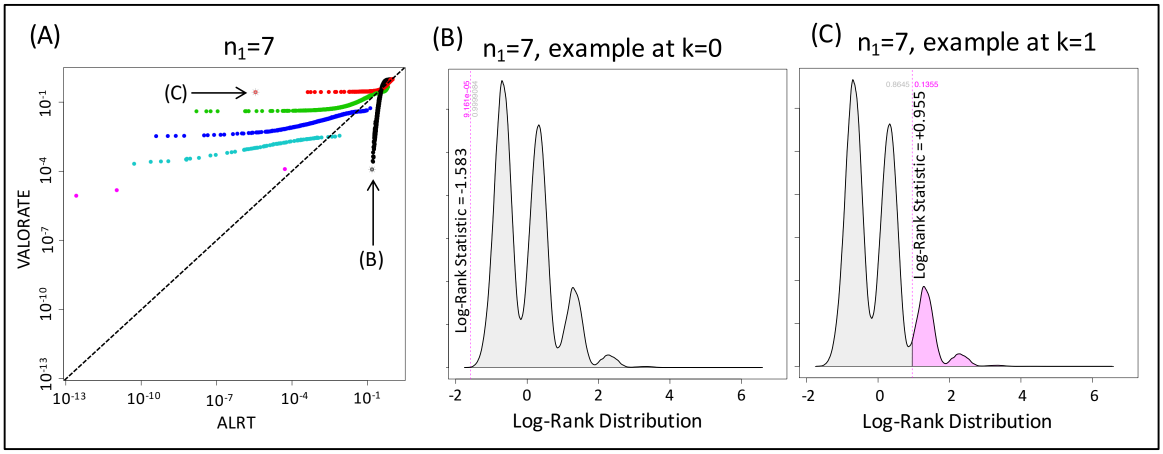

Supplementary Figure 4. Examples of differences in the p-value estimation. (A) Shows the estimated p-values from the ALRT (horizontal axis) and VALORATE (vertical axis) for a simulation having *n=100, d=10,* and *n1=7* (as in Supplementary Figure 3). Colors correspond to the number of co-occurrences (black=0, red=1, green=2, blue=3, cyan=4, magenta=5, and 6 and 7 were not observed in this sampling). "*" at the bottom right (black) and top left (red) marks two extreme cases shown in (B) and (C) respectively. (B) The estimated p-value using VALORATE of 1.8×10^{-4} which was estimated by the ALRT as p=0.15. (C) The estimated p-value using VALORATE of 0.27 which was estimated by the ALRT as $p=3.5 \times 10^{-6}$.

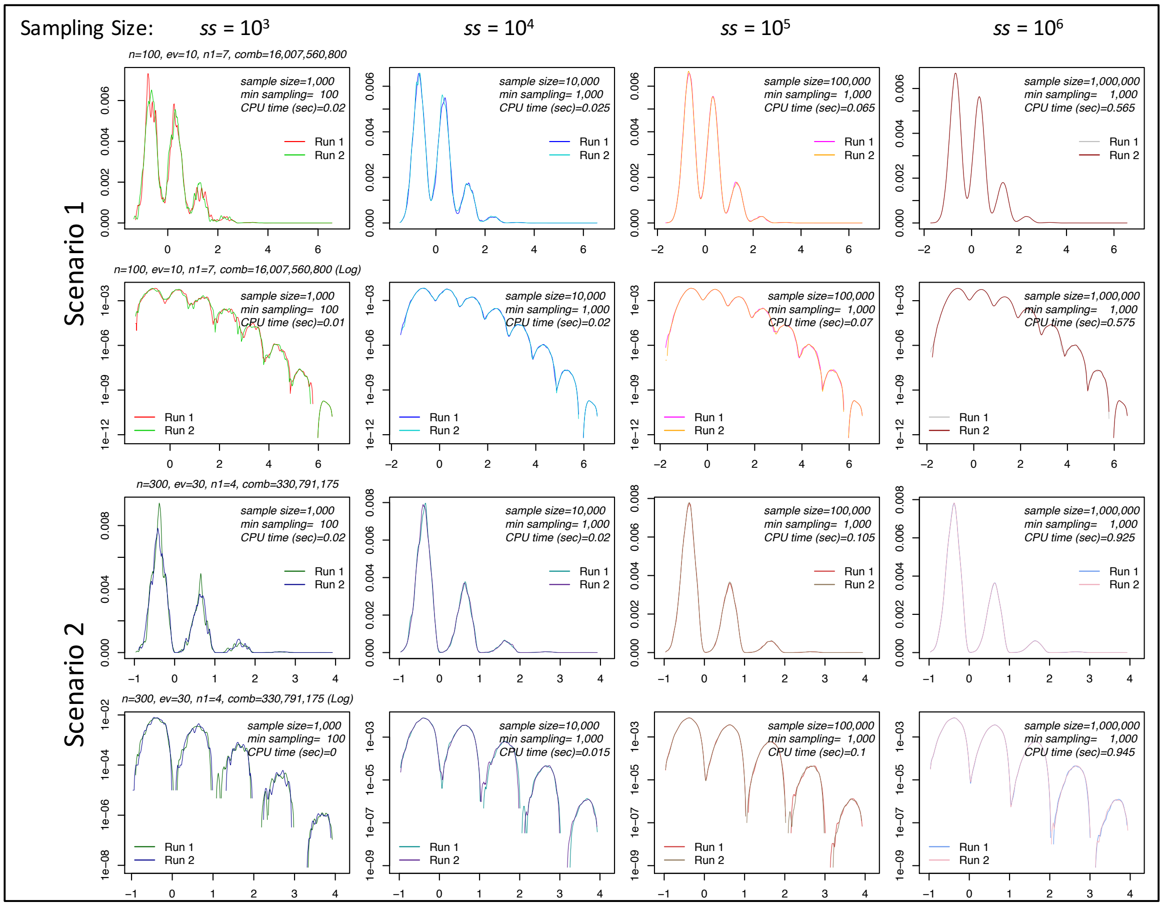

Supplementary Figure 5. Precision of VALORATE at different values of sampling size. Two parameters sets (scenarios) were used. The top 2 rows show simulations at *n=100, d=10 (ev), n_1=7* and the 2 bottom rows at *n=300, d=30 (ev), n_1=4*. Columns show different values of the sampling size parameter (*ss*) corresponding to 10^3, 10^4, 10^5, and 10^6. Each panel shows two runs in different colors. Row 1 and 3 correspond to raw scale whereas rows 2 and 4 correspond to logarithm base 10 scale to highlight low-density regions.

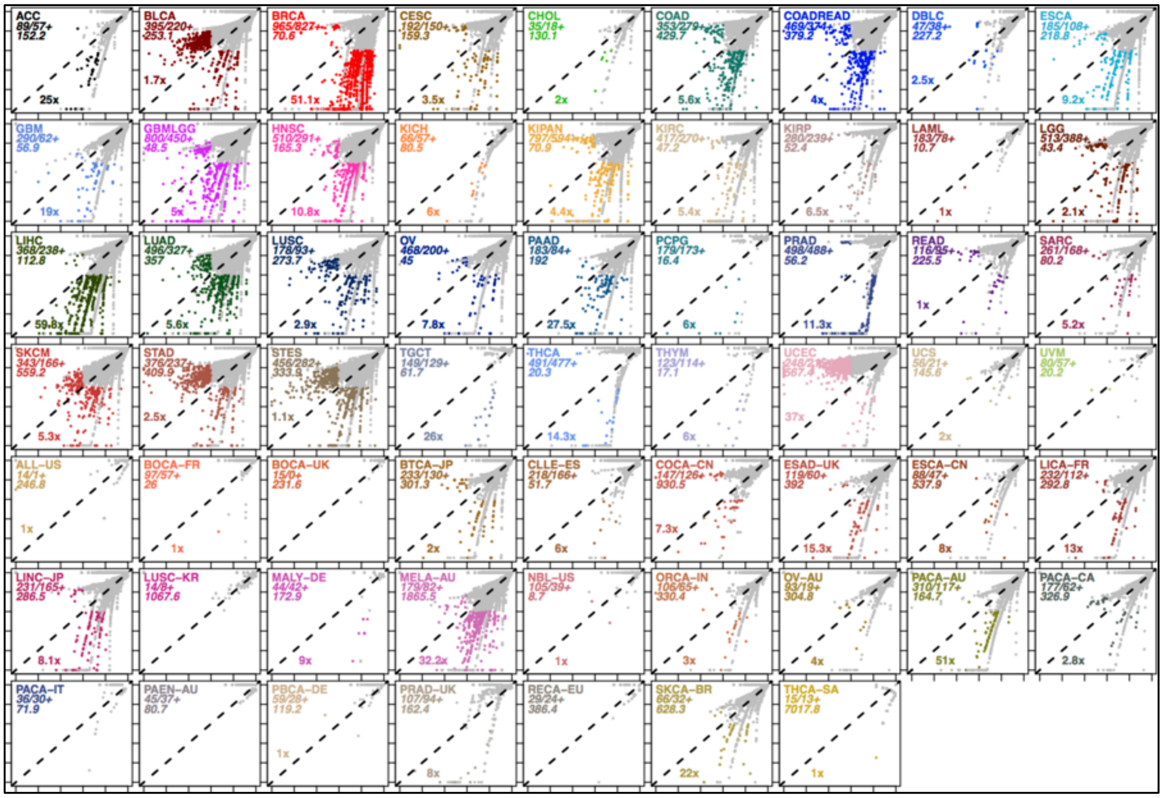

Supplementary Figure 6. Differences of p-value estimations across cancer types. Each panel shows a cancer type, the samples used, the number of censored samples, the average number of mutations per sample, and the p-value estimations for VALORATE (horizontal axis) and the ALRT (vertical axis). Each dot corresponds to a gene in the dataset. Only genes whose p-value < 0.01 in any test and having 4 or more mutations are colored.

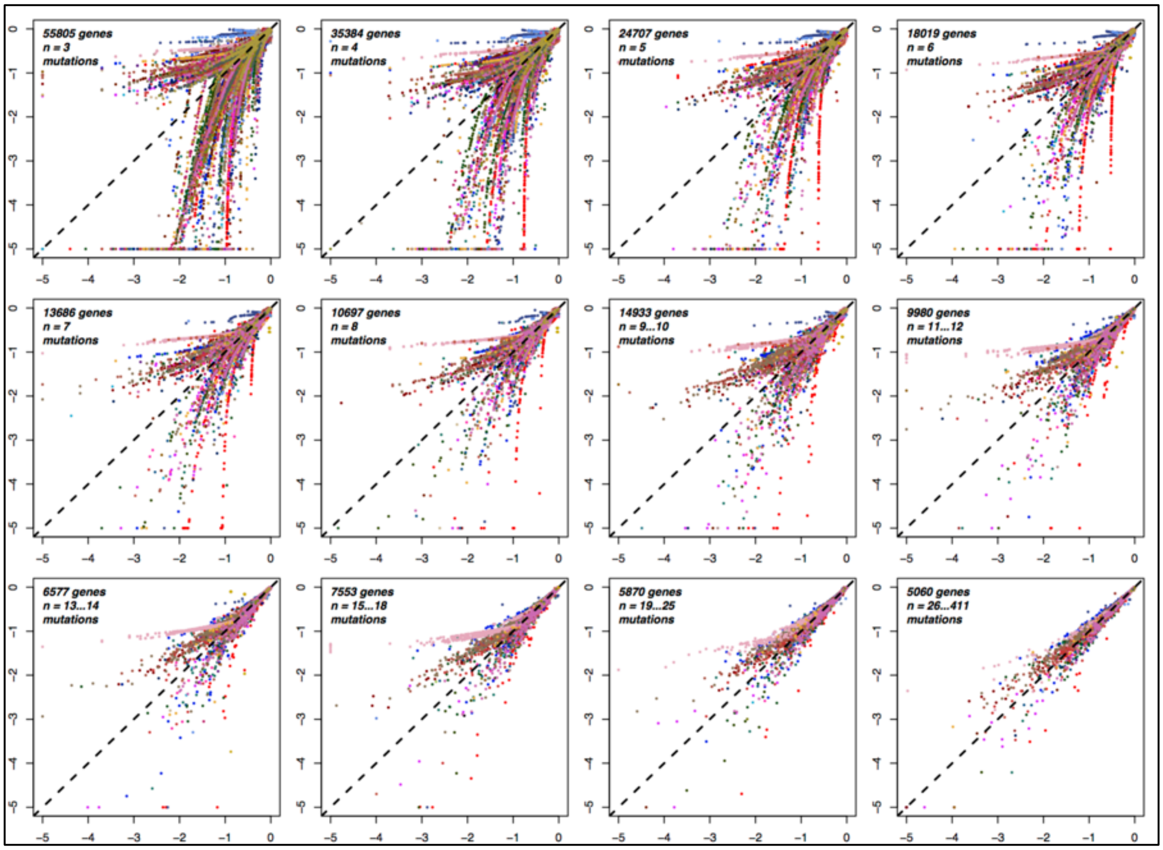

Supplementary Figure 7. Differences of p-value estimations along a number of mutations. Each panel shows the p-value estimated in VALORATE (horizontal axis) and the ALRT (vertical axis) for the specified number of samples mutated (from 3 to more than 25). Colors correspond to cancer types.

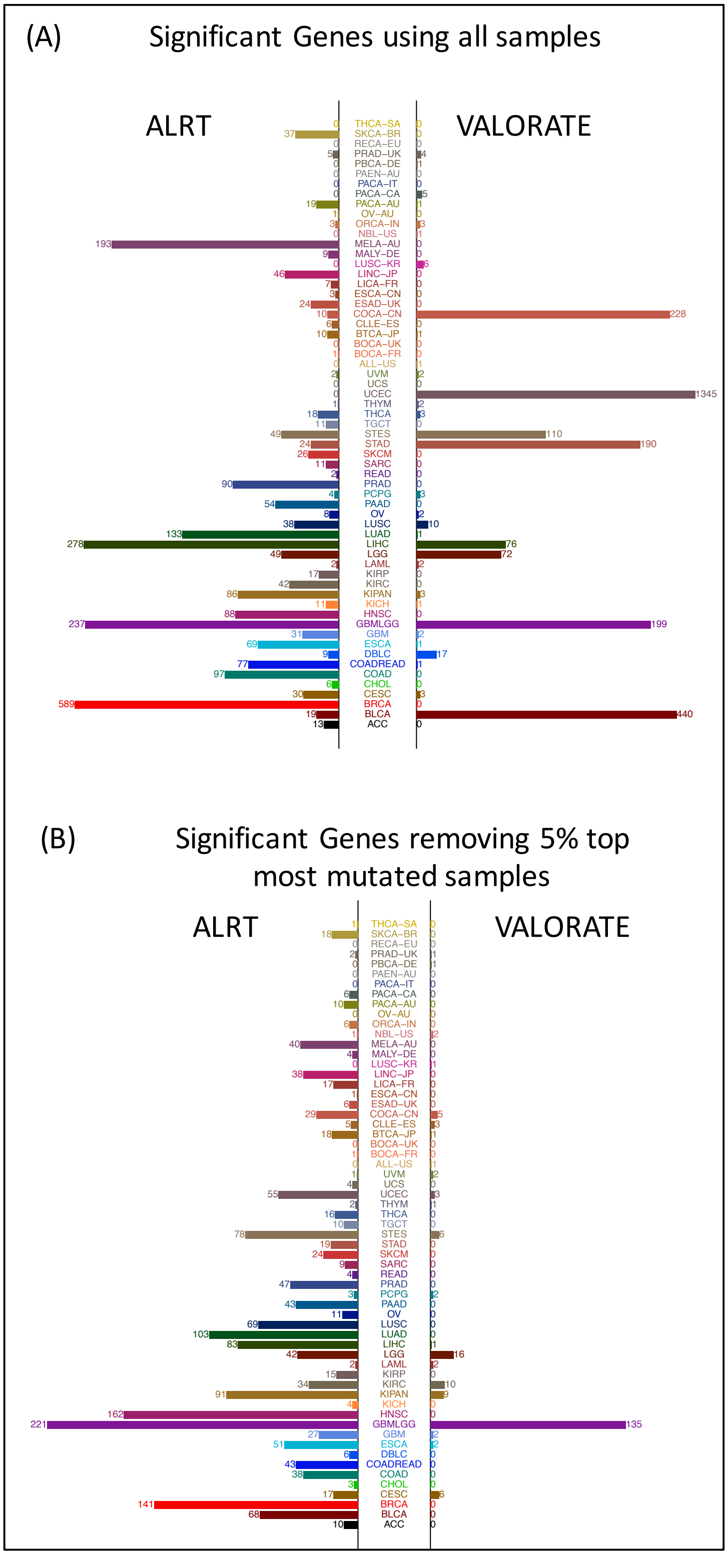

Supplementary Figure 8. Number of significant genes at FDR=0.333 across cancer types. (A) Significant genes using all samples in VALORATE and the ALRT. (B) Significant genes after removal of top 5% most mutated samples.

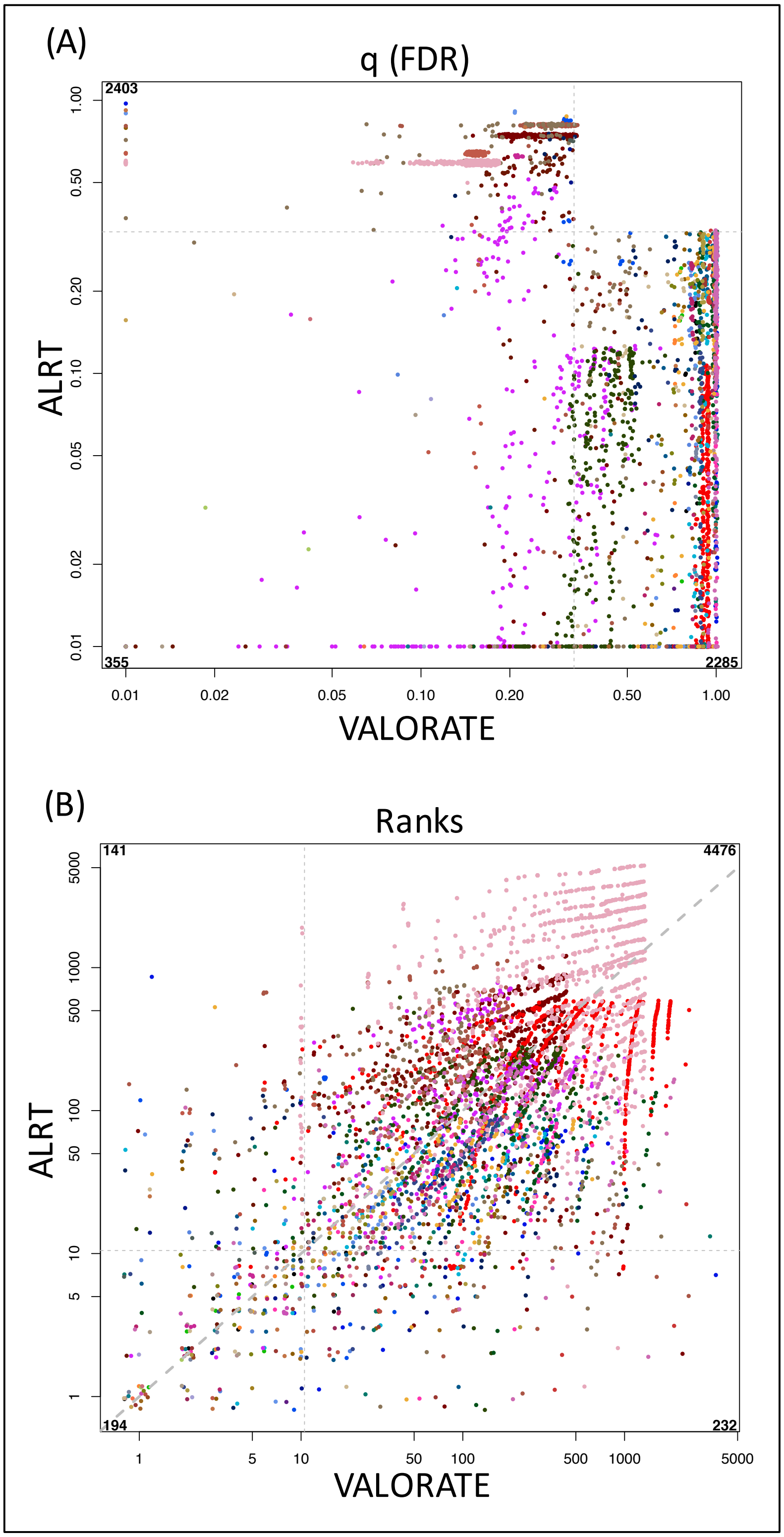

Supplementary Figure 9. Comparison of significant and top genes. (A) q-value of genes significant at q-FDR < 0.333 and p < 0.05 in VALORATE (horizontal axis) or in the ALRT (vertical axis). (B) Ranks of genes in (A).

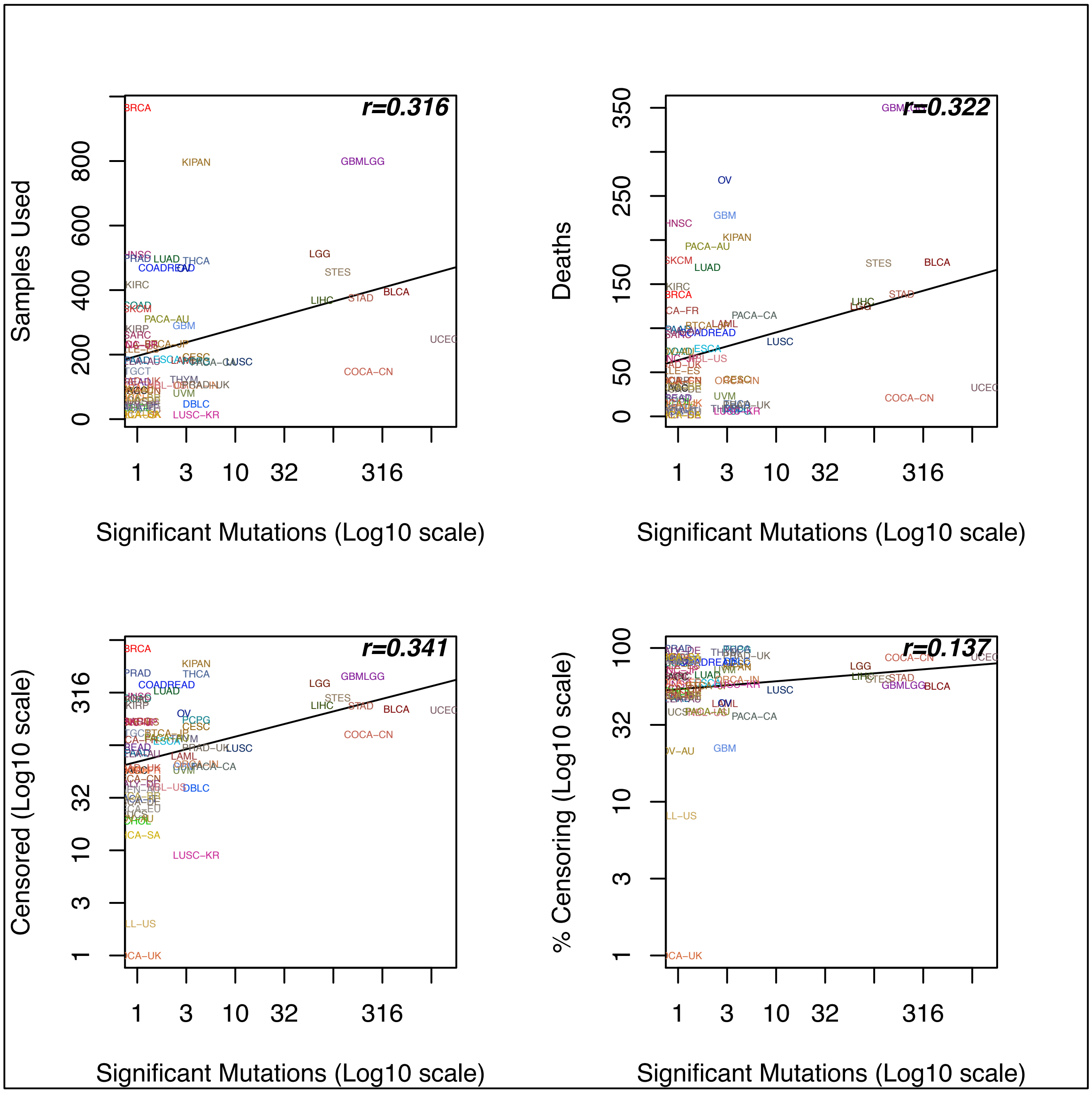

Supplementary Figure 10. Association of the number of significant genes with the numbers of samples. Association to (A) the number of samples used, (B) deaths, (C) censored, and (D) percentage of censoring.

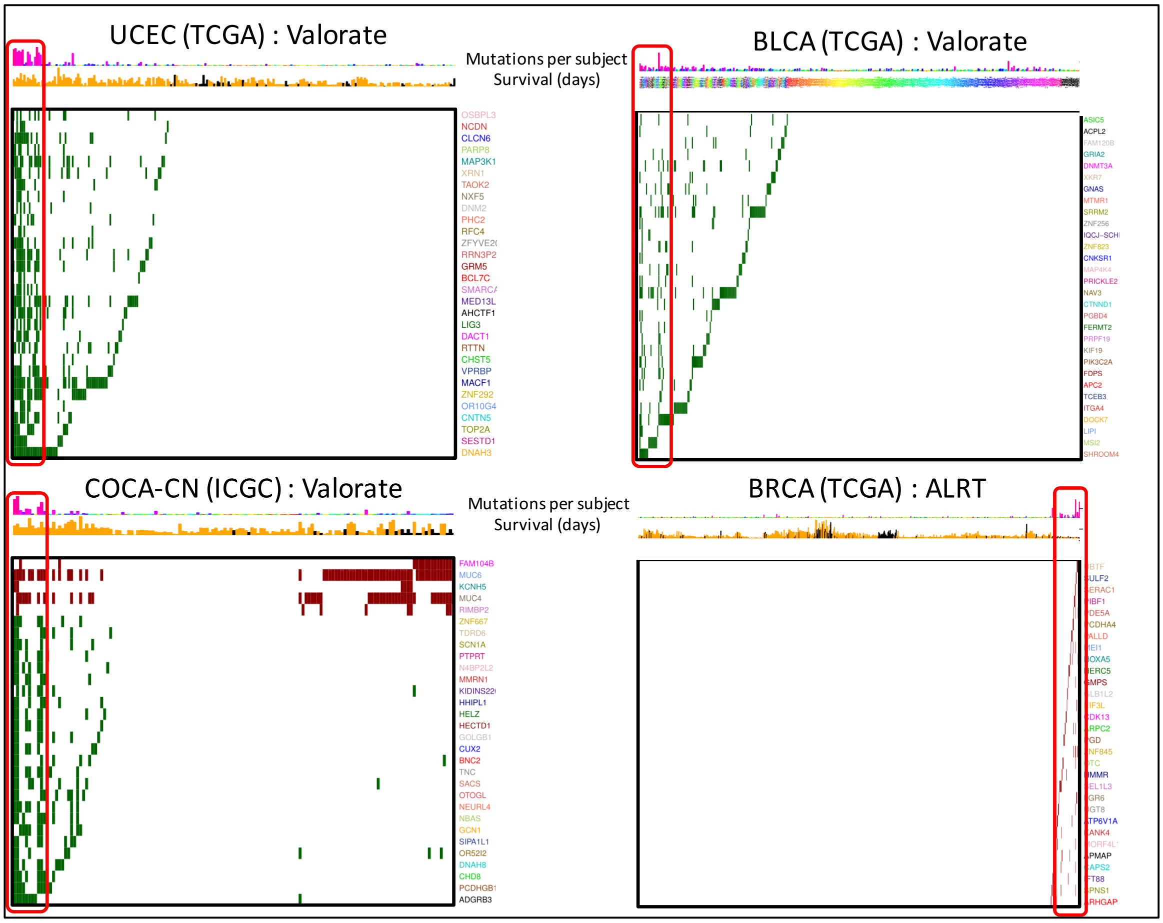

Supplementary Figure 11. Association of the number of significant genes with hypermutated of samples. The panels show the top 30 most significant genes (having lowest p-values) in the vertical axis and samples in the horizontal axis. The number of mutations per subject and the survival days is also shown on top of each panel. The ordering of columns corresponds to the significance and whether the gene was associated with low risk (green) or high risk (red). The columns were ordered by the number of mutations in low and high risk within the genes shown.

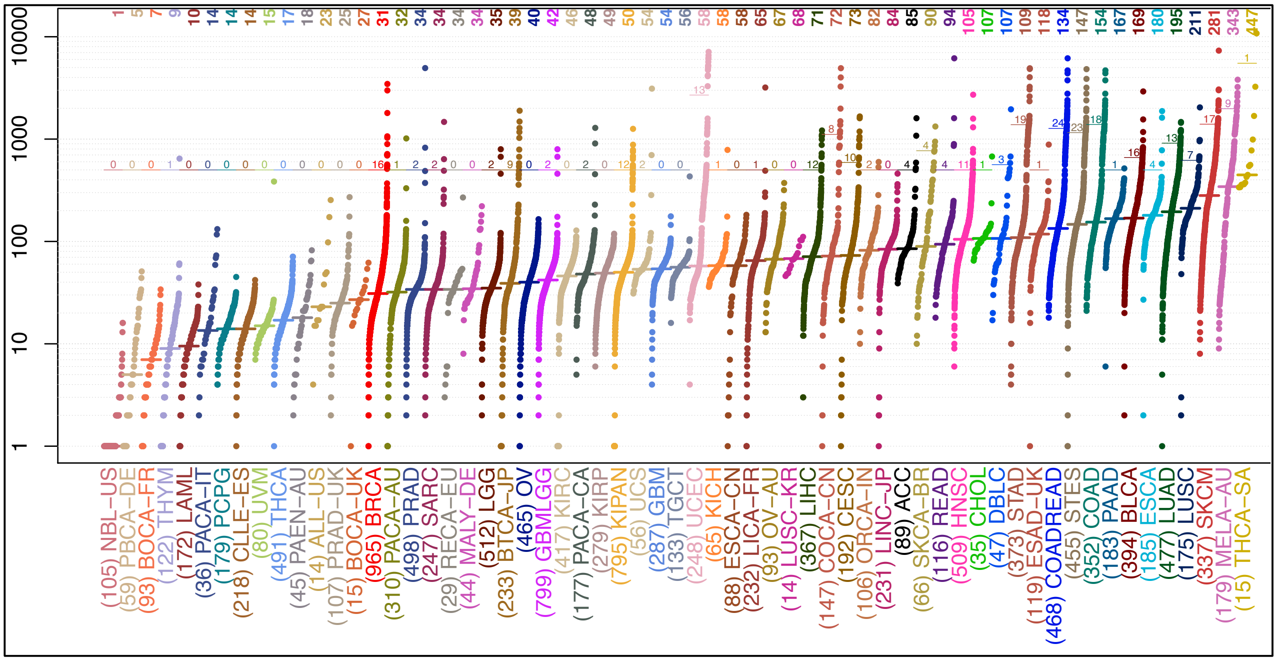

Supplementary Figure 12. Number of hypermutated samples removed along with mutated genes per sample and cancer type. Each dot corresponds to a sample within a cancer type (horizontal axis). Cancer types ordered by the median of the number of mutated genes. The number of samples of each cancer type is shown in parenthesis. The number of 'hypermutated' samples removed are shown above the line marking the cut-off used. For most cancer types, a 500 cut-off value was used.

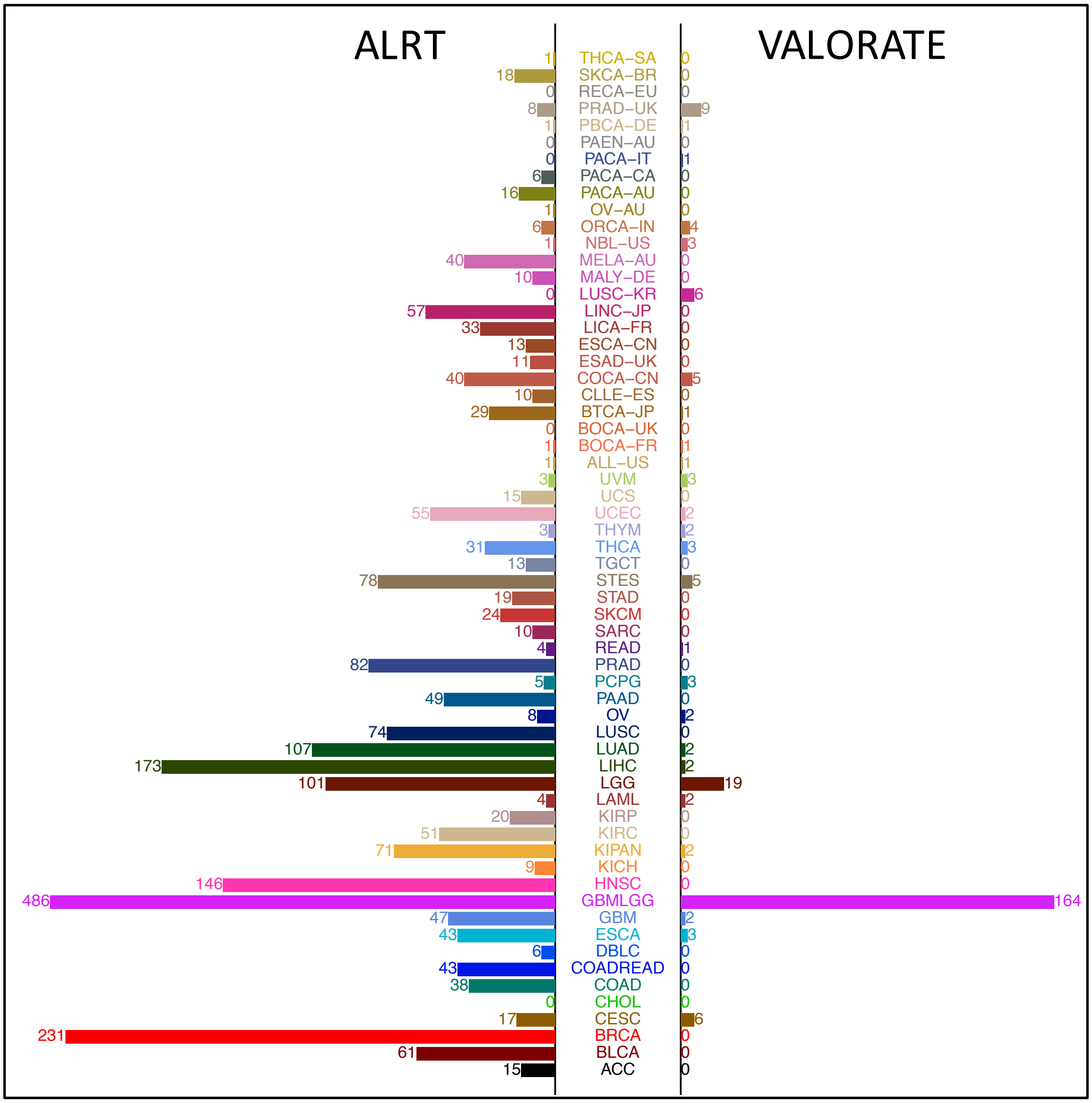

Supplementary Figure 13. Number of significant genes per cancer type after removal of hypermutated samples.

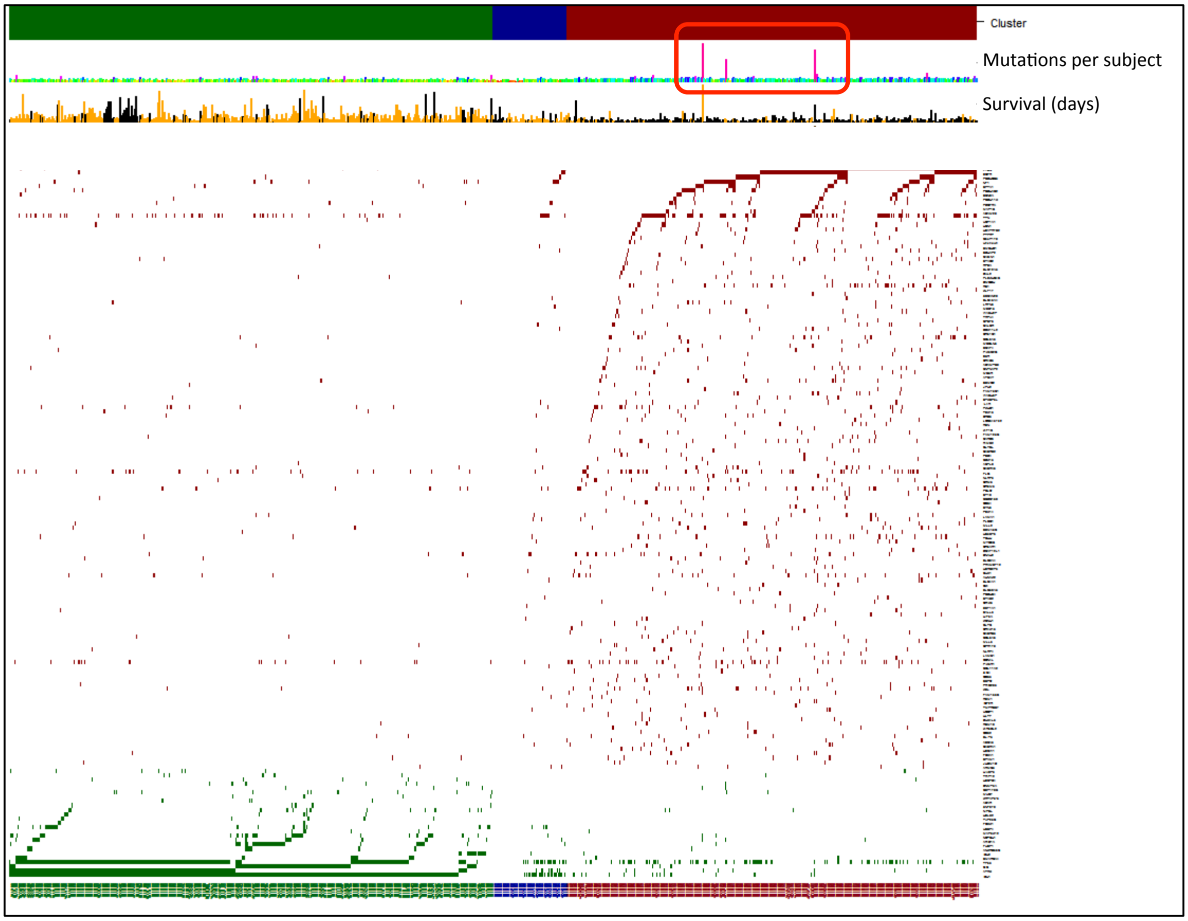

Supplementary Figure 14. Significant genes using VALORATE in gliomas are not related to most mutated samples.

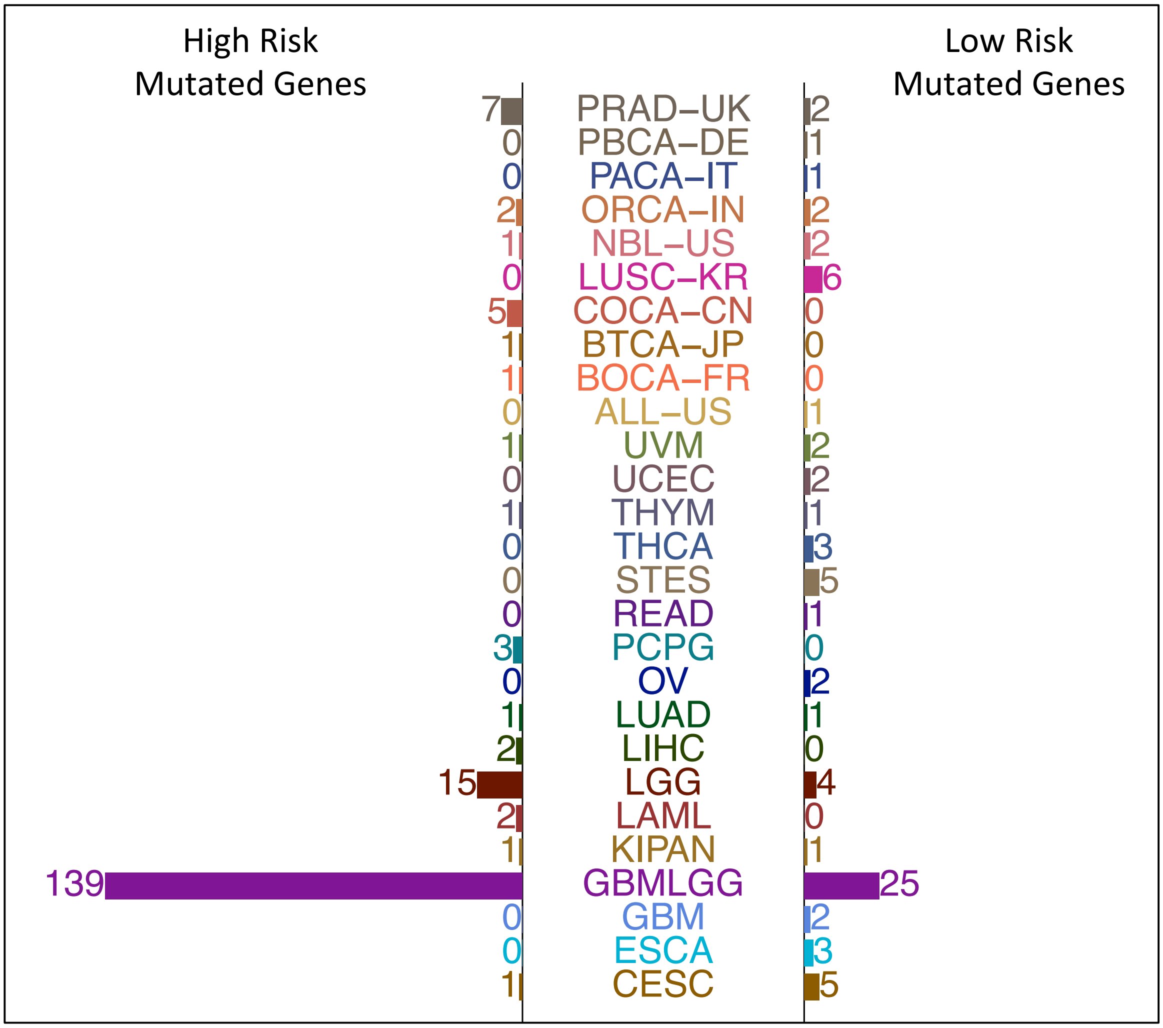

Supplementary Figure 15. Risk group associated with significant genes using VALORATE.

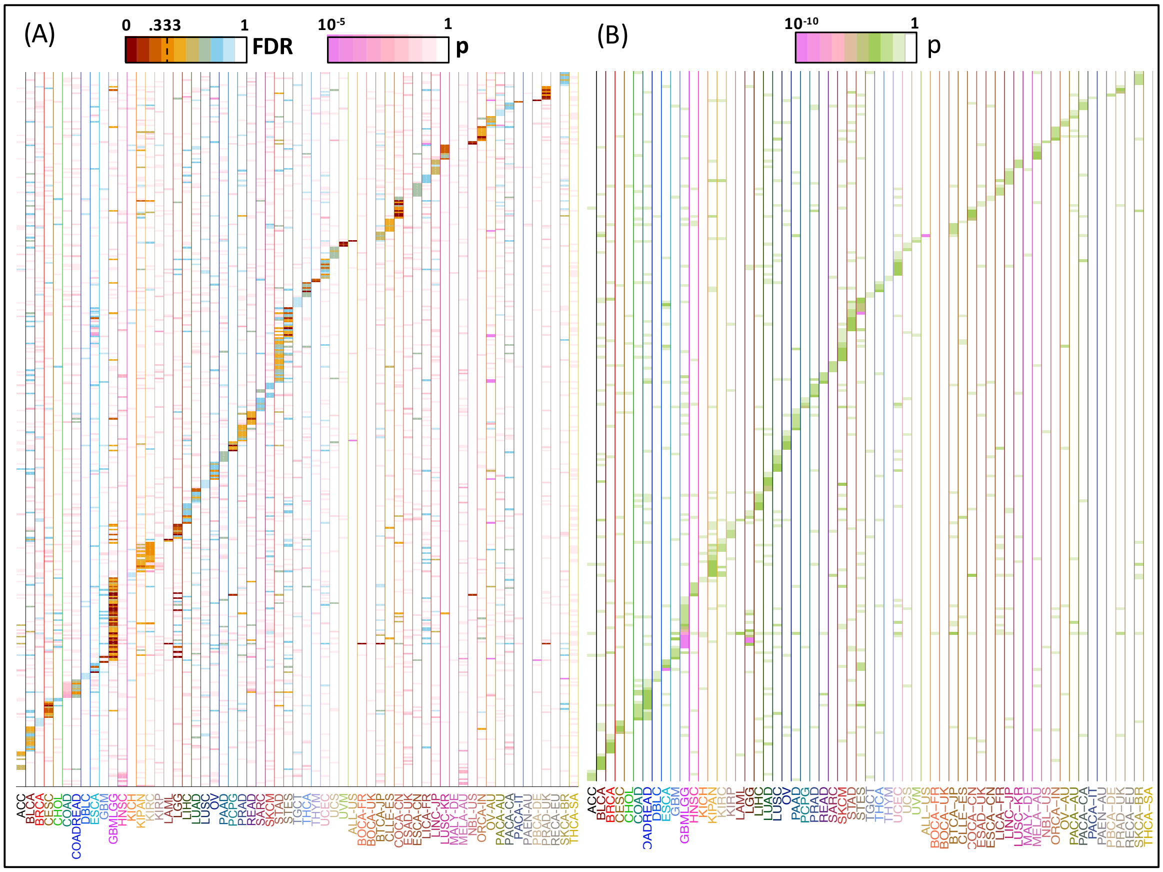

Supplementary Figure 16. Top genes seem cancer-type specific. (A) Significant genes relaxing the FDR cut-off to FDR < 0.999, $p < 0.05$, and maximum 10 genes per cancer type. (B) The p-value of the top 5 genes per cancer type.

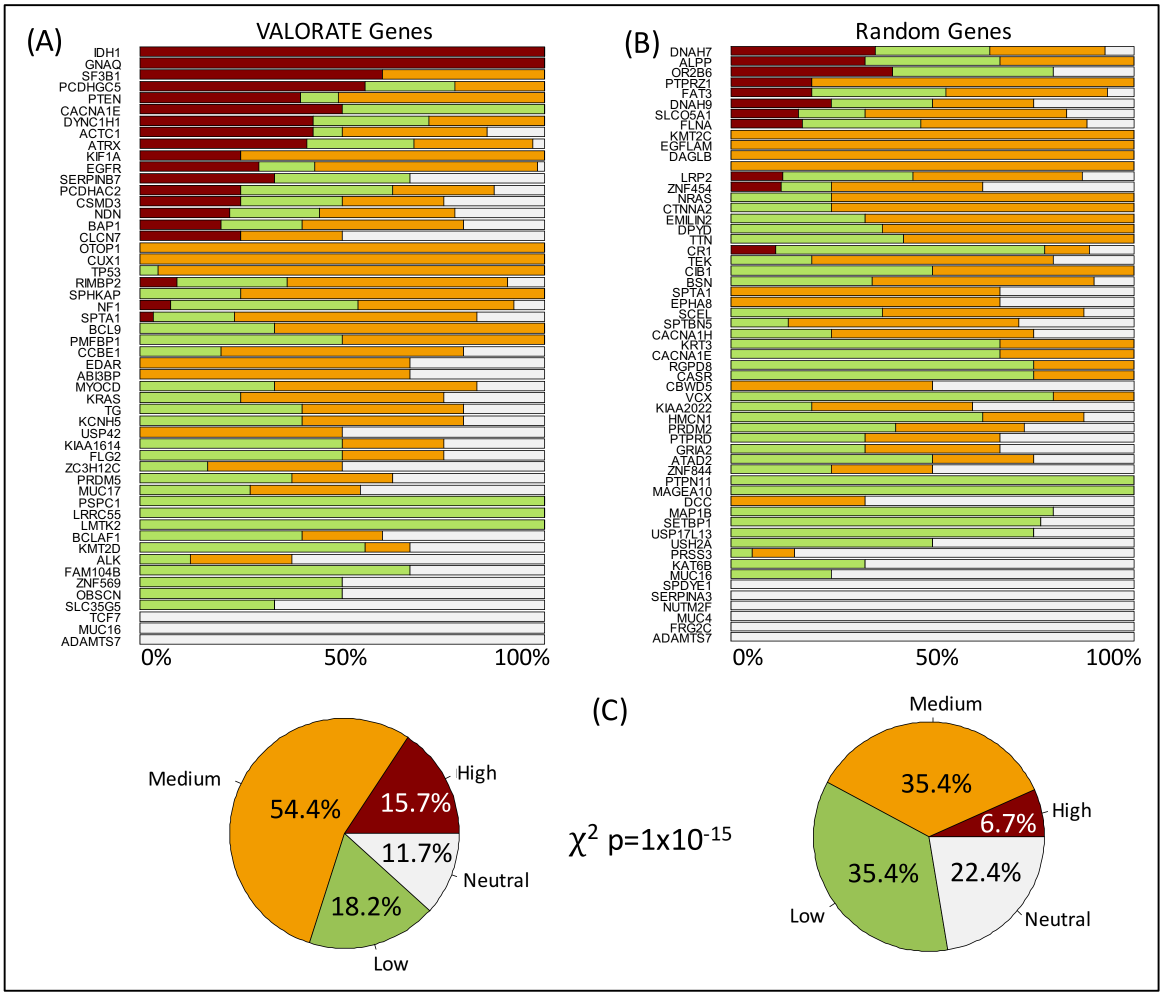

Supplementary Figure 17. Functional impact of the mutations in significant genes. (A) Functional impact of the mutations in significant genes. (B) Functional impact of random genes having a similar number of mutations within the same cancer types. The annotations were obtained from MutationAssessor. (C) Statistical analysis of the differences in functional impact.

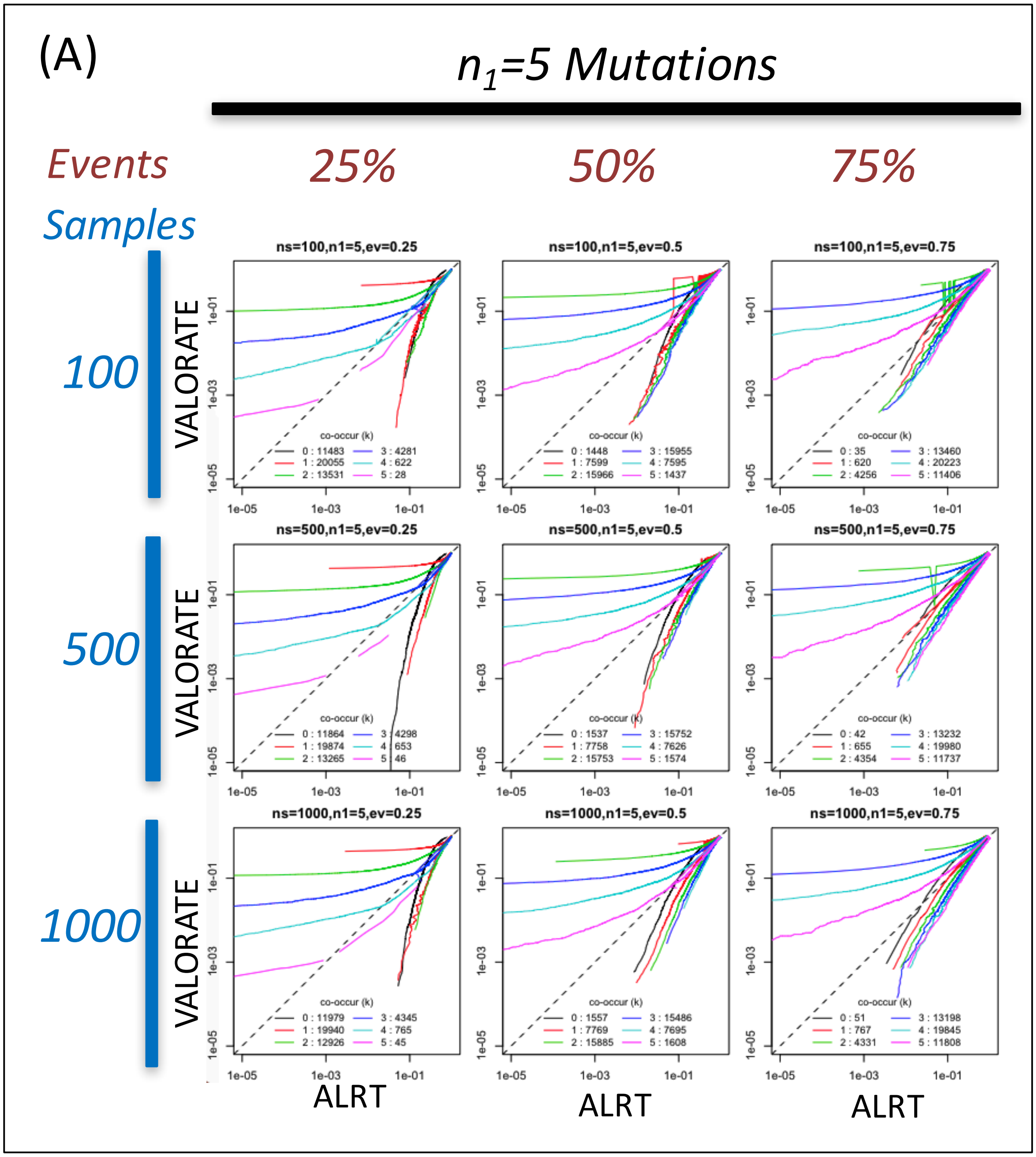

(Supplementary Figure 18)

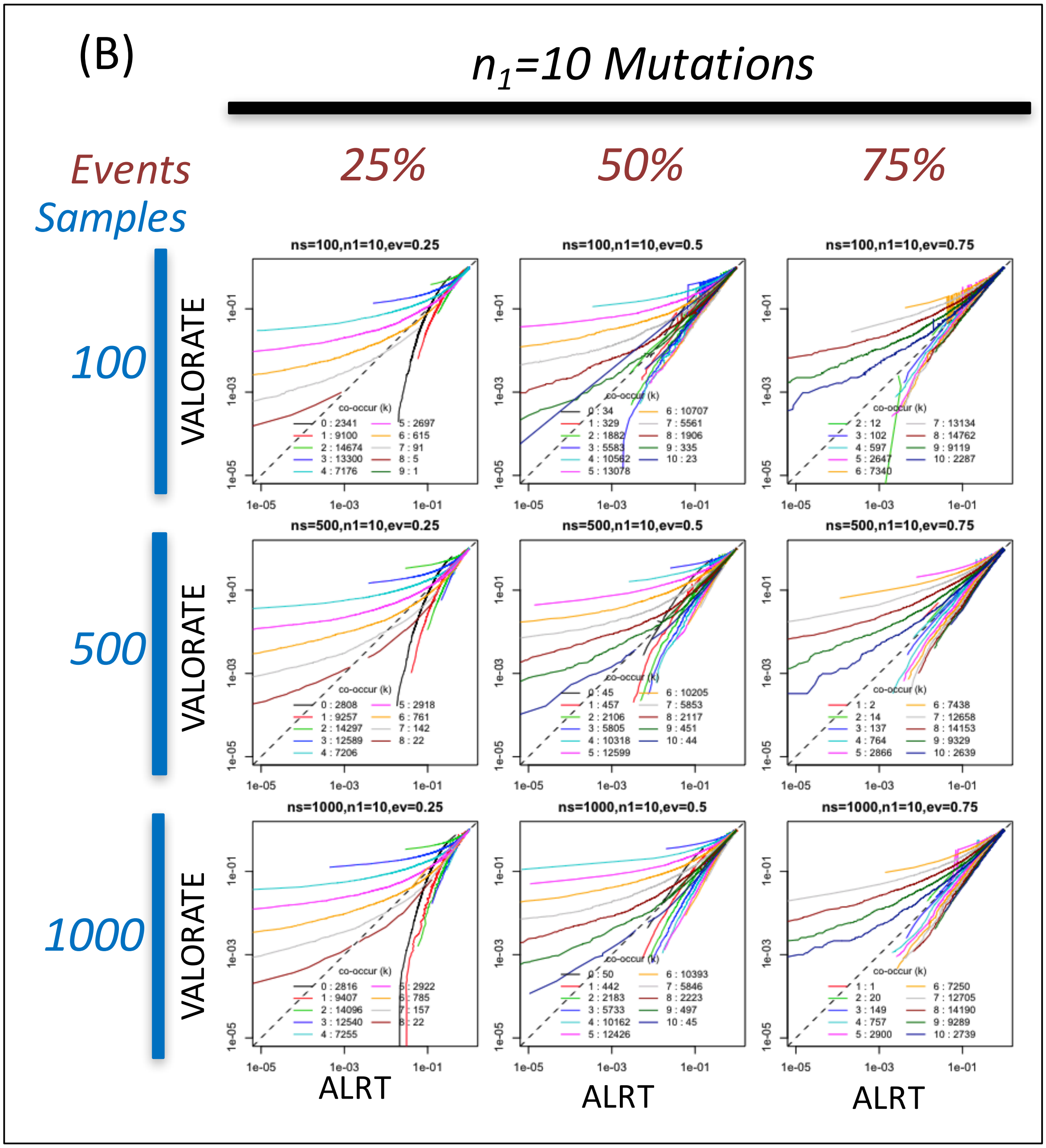

Supplementary Figure 18. Comparisons of p-value estimations in several settings. To clarify the tendencies in all panels, the points for a value of *k* that were placed above the diagonal were joined used a line, which was then smoothed. A similar line was used for the points below the diagonal. (A) For 5 mutations in settings of 25%, 50%, and 75% of events in 100, 500, and 1000 samples. (B) For 10 mutations in same settings than (A). Note that patterns depend on % of events and mutations and not on the number of samples.

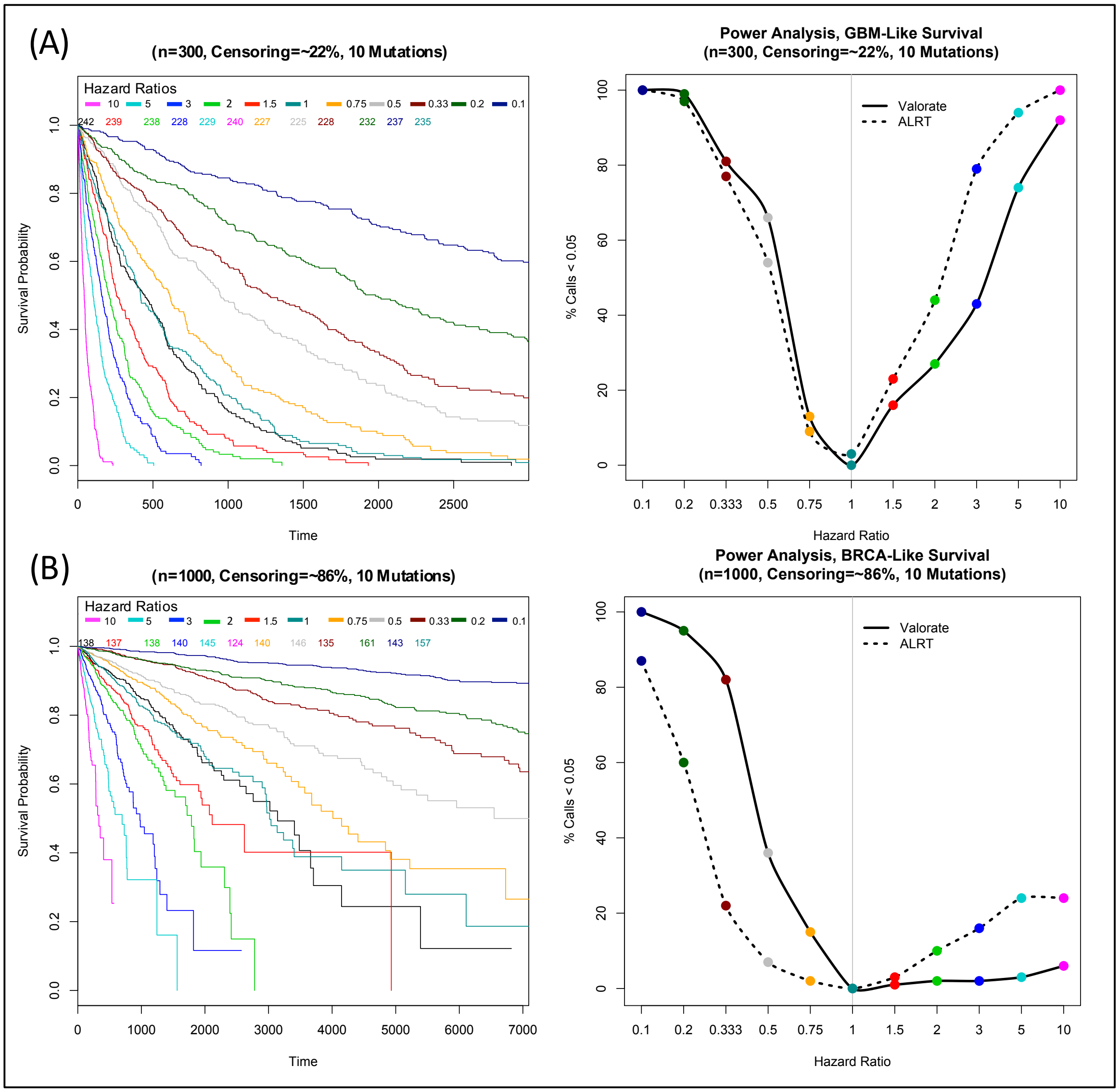

Supplementary Figure 19. Power analysis in two scenarios. The figure shows the results of the simulations for data similar to Glioblastoma (GBM) in (A) and for Breast cancer (BRCA) in (B). The panels at left show the simulated populations at different hazard ratios while the panels at right show the power analyses. 100 simulations were performed for each hazard ratio tested. Each simulation used all subjects from the original fitting (black Kaplan-Meier curve) and added 10 random subjects from the tested curve that were used as the 'mutated' subjects. For VALORATE, we used 100,000 for sampling size. The simulations were performed using a uniform hazard over time for all subjects. Censoring was randomly determined at the moment of simulated death multiplying the death time by a random uniform factor between 0.5 and 1 to generate the censored follow-up time. The parameters of our simulations were adjusted to fit the observed data in GBM in (A) and BRCA in (B).

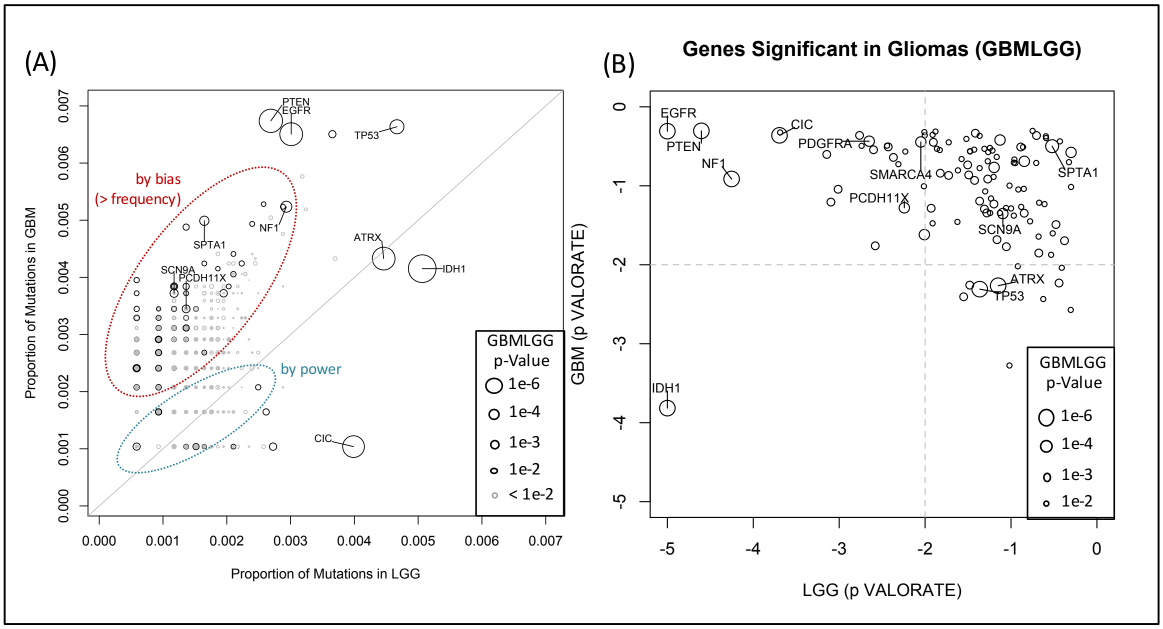

Supplementary Figure 20. Comparison of the mutation frequency and significance of genes between Gliomas and Glioblastoma and Low-Grade Gliomas. (A) shows the proportion of samples for Glioblastoma (GBM) in the vertical axis and for Low-Grade Gliomas (LGG) in the horizontal axis. Colored ellipses denotes p-value estimations likely affected by differences in mutation frequency (red) and those gaining power due to higher number of samples (blue). The size of the bubble is related to the p-value for Gliomas (GBMLGG). Note that smaller bubbles are closer to the diagonal. Gray denotes non-significant (at FDR < 0.333). Top genes in gliomas (GBMLGG) are labelled. (B) shows a comparison of the p-value in logarithm base 10 scale of significant genes in Gliomas.
